# Timely integration of palliative care into oncology in hospitals in the Netherlands: a feasibility study

**DOI:** 10.1186/s12913-025-13199-2

**Published:** 2025-09-30

**Authors:** Carly S. Heipon, Linda Brom, Wendy M. van der Deure, Irene Dingemans, Olaf P. Geerse, Saskia Lauer, Filip Y. F. L. de Vos, Yvette M. van der Linden, Anna K. L. Reyners, Natasja J. H. Raijmakers

**Affiliations:** 1 Department of Research & Development, Netherlands Comprehensive Cancer Organisation (IKNL), Utrecht, The Netherlands; 2 Department of Internal Medicine, Groene Hart Hospital, Gouda, The Netherlands; 3 Dutch Federation of Cancer Patients Organisations (NFK), Utrecht, The Netherlands; 4 Department of Medical Oncology, Amsterdam UMC, Amsterdam, The Netherlands; 5 Cancer Centre Amsterdam, Cancer Treatment and Quality of Life, Amsterdam, The Netherlands; 6 Department of Medical Oncology, Wilhelmina Medical Centre, Assen, The Netherlands; 7 Department of Medical Oncology, University Medical Centre Utrecht, Utrecht, The Netherlands; 8 Department of Radiotherapy, Leiden University Medical Centre, Leiden, The Netherlands; 9 Centre of Expertise in Palliative Care, Leiden University Medical Centre, Leiden, The Netherlands; 10 Department of Medical Oncology, University Medical Centre Groningen, University of Groningen, Groningen, The Netherlands; 11 Centre of Expertise in Palliative Care, University Medical Centre Groningen, University of Groningen, Groningen, The Netherlands

**Keywords:** Palliative care, Oncology, Timely integration, Pilot study, Feasibility

## Abstract

**Background:**

Timely integration of palliative care has numerous benefits for patients with incurable cancer. Based on a recent national Delphi study on the timely integration of palliative care in oncology, three recommendations were formulated regarding 1) advance care planning (ACP), 2) routine symptom monitoring during the last year of life, and 3) involving the Specialist Palliative Care Team (SPCT) during the last three months of life. This pilot study aimed to assess the feasibility of these recommendations in the Dutch context.

**Methods:**

Four Dutch hospitals implemented these recommendations for three months. Feasibility was assessed in three ways. First, the extent to which the recommendations were applied was assessed by analysis of electronic medical records (EMRs) of 542 patients with incurable cancer. Second, the extent to which clinicians (n = 27) found the recommendations applicable was assessed using a questionnaire including the Measurement Instrument for Determinants of Innovations (MIDI) and self-administrated questions. Last, patients’ experiences (n = 70) were assessed using the EORTC IN-PATSAT and self-administrated questions regarding using the recommendations.

**Results:**

The recommendation on ACP was applied in 49% of eligible patients and the recommendation on symptom monitoring and SPCT in 58%. Most clinicians agreed that all three recommendations were important to achieve timely integration of palliative care (85%). The majority reported being able to conduct ACP discussions (78%), to consider involving the SCPT in case of complexity (73%), and to offer SPCT consultation in case of a life expectancy of ≤ 3 months (68%). A minority reported being able to pay attention to symptom monitoring across the four dimensions (42%) and to record outcomes in the EMR (19%). Patients who received care according to the recommendations were equally satisfied with care compared to those who received standard care, except when symptoms of social problems were monitored; these patients were more satisfied with care, respectively 83.1(SD 18.7) vs 71.2(SD 20.1).

**Conclusions:**

Implementing the recommendations for timely integration of palliative care in daily clinical oncology practice seems feasible. The key is to address practical issues, including information exchange about ACP and symptom management among all involved clinicians.

**Supplementary Information:**

The online version contains supplementary material available at 10.1186/s12913-025-13199-2.

## Introduction

Improved and novel cancer treatments enable patients with incurable cancer to live longer, giving rise to palliative care needs [1]. However, despite these medical advances, cancer remains a leading cause of death worldwide [2]. It has also been predicted that by 2040 cancer will be one of the main drivers of the increased need for palliative care [3]. The WHO defined palliative care as ‘an approach that improves the quality of life of patients and their families facing life-threatening illness, through the prevention and relief of suffering through early identification and impeccable assessment and treatment of pain and other problems, physical, psychosocial and spiritual’[4].

A substantial body of literature has highlighted the importance of the timely integration of palliative care into oncology care for patients. It has been shown to improve patients’ quality of life [5–9], reduce depressive symptoms [10, 11] and caregiver burden [12], increase satisfaction with care [13], decrease the chance of potentially inappropriate end-of-life care [14] and even prolong survival [5, 15]. A systematic Cochrane review showed small effect sizes of early palliative care on patient outcomes, and emphasizes the need for further research to assess the effect of early palliative care [16]. These findings have led to in international guidelines, recommendations, criteria, and statements for early palliative care integration [17–20]. Thirteen major indicators of integrated palliative care and oncology for patients with incurable cancer were identified, such as an interdisciplinary hospital palliative care team, routine symptom screening in the oncology outpatient clinic, routine documentation of advance care plans, and early referral to specialist palliative care [21]. In the Dutch healthcare system, timely integration is sought through the application of the generalist-specialist model. In this model, all clinicians are considered palliative care generalists and provide palliative care as part of standard care, based on their basic medical skills. They are supported by palliative care specialists when needed. Palliative care specialists are (often) additionally trained in palliative care and organised in a specialist palliative care consultation team (SPCT). Clinicians can consult the SPCT for peer-to-peer consultation or refer patients to the team.

A recent Dutch Delphi study identified essential elements of the timely integration of palliative care for patients with incurable cancer [22]. Based on these elements, three recommendations for advance care planning, routine symptom monitoring and referral to the SPCT were formulated, including guidance on when, how and by whom these recommendations should be implemented. However, it is unclear whether applying these recommendations is feasible in everyday oncology practice. Therefore, we aimed to assess the feasibility of these recommendations by pilot testing them in the Dutch clinical context. Three research questions were addressed: (1) to what extent were clinicians able to apply the recommendations in daily clinical practice?, (2) to what extent are the recommendations applicable to clinicians?, and (3) how do patients assess the care they received?

## Methods

### Study design

**Fig. 1:**
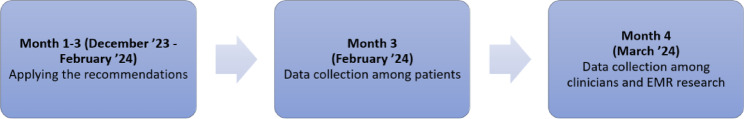
Research timeline by month

The pilot study to assess the feasibility of the recommendations was conducted between November 2023 and March 2024. The applicability of the recommendations according to clinicians was assessed in month 4 (post-measurement) via a questionnaire that included the Measurement Instrument for Determinants of Innovations (MIDI) (Supplementary material 1) [23] and additional self-administrated questions. The electronic medical records (EMR) of patients with incurable cancer who were clinically admitted or seen as outpatients during month 3 of the pilot were assessed to analyse the extent to which clinicians could apply the recommendations (Fig. 1). During the last month of the pilot period (month 3) patients received a questionnaire to assess their experiences with the received care using the EORTC IN-PATSAT32 and self-administrated questions (Supplementary material 2).

The pilot consisted of implementing the following three recommendations for the timely integration of palliative care:

1. Offer advance care planning (ACP)^1^ to all patients with incurable cancer and conduct a (follow-up) ACP discussion in the event of increased symptom burden, a life expectancy of < 1 year (identified using the Surprise Question (SQ) ‘Would I be surprised if this patient dies within 12 months?’) and/or a change in line of treatment. Record the outcomes of these discussions in the EMR and share them with the primary care physician or other involved institutional physicians.
2. Monitor symptoms and perform symptom management routinely across the four dimensions of Quality of Life (QoL) (physical, psychological, social and spiritual) in patients with incurable cancer with a life expectancy of < 1 year. Record the outcomes of symptom monitoring and corresponding treatment decisions in the EMR.
3. Consider involving the SPCT in complex cases, such as when there is symptom burden on multiple dimensions, and/or if the patient requests their involvement. Offer a consultation with the SPCT to patients with incurable cancer with life expectancy of < 3 months (identified using the adapted Surprise Question (SQ) ‘Would I be surprised if this patient dies within 3 months?’).

As this was an exploratory feasibility study, the process of applying the recommendations was kept close to the workflows of the pilot hospitals. No standardised training or protocol was imposed; instead, hospitals used their own existing methods to apply the recommendations during the three-month period. Therefore, the implementation of the three recommendations was tailored to each hospital (Supplementary Table 1). After 1.5 months of applying the recommendations an online meeting was scheduled with each pilot hospital to evaluate and discuss the progress. The Strengthening the Reporting of Observational Studies in Epidemiology (STROBE) and Checklist for Reporting of Survey Studies (CROSS) checklists were used for reporting [25, 26].

### Setting

The four pilot hospitals were recruited using the expert panel from the previous Delphi study on elements of integrated care [22]. All clinicians involved were asked whether their department would want to participate. The four participating hospitals (one academic and three non-academic) included three medical oncology and one pulmonary oncology department. Three pilot hospitals implemented the intervention in both the in- and outpatient setting, one hospital only in the outpatient setting.

### Measures

#### Extent to which clinicians applied the recommendations

To assess the extent to which the recommendations were being applied, a cross-sectional EMR analysis was performed of all patients with incurable cancer who were admitted to the hospital and/or seen in the outpatient clinic during month 4 of the pilot. To enhance representativeness and limit bias, the EMRs were consecutively analysed by one or more assessors. These assessors were clinicians and assigned by the pilot hospital. The following information was extracted from the EMRs: patient characteristics (age, gender, tumour type), care setting (in- or outpatient), if the patient had already had an ACP discussion in the year prior to the study. If so, it was noted whether the outcomes were shared with the general practitioner (GP) or involved institutional clinicians. Assessors then indicated, based on their own clinical knowledge, whether they would be surprised if the patient died within 12 months. If answered negatively, it was recorded whether outcomes of symptom monitoring and corresponding treatment decisions were documented. Assessors also indicated whether they would be surprised if the patient died within 3 months. If not, it was recorded whether the patient was offered a consultation with the SPCT. We used a one-year timeframe for ACP discussions to include patients who had had ACP discussions before the start of the pilot and for whom there was no indication for another ACP discussion during the pilot. Therefore, we examined whether patients had had an ACP discussion in the year prior to the pilot study; if so, this would suggest that the recommendation was already being applied. The number of medical records to be analysed was set to a maximum of 150 patients per hospital. To ensure face validity, the results of the EMR analysis were discussed with the participating clinicians at each of the pilot hospitals. Local data extraction and management were carried out by the clinical trial offices affiliated with each hospital, in accordance with local protocols and access procedures. No central validation or verification of the individual records was performed.

#### Applicability of recommendations from clinicians’ perspective

To evaluate the applicability of the recommendations according to clinicians who had implemented them, all clinicians received a questionnaire in month 4. This questionnaire included the MIDI instrument. The MIDI instrument has 29 items and is divided into four categories of determinants associated with either the innovation, user, organisation and, socio-political context. Clinicians received the complete MIDI instrument, but only the determinants associated with the user (11 items) are reported since these were most relevant to determine clinicians’ perspectives. The MIDI uses a 5-point Likert scale response format (‘strongly agree’, ‘agree’, ‘do not agree’, ‘do not disagree’, ‘disagree’, ‘strongly disagree’) [23]. The only exception was the question on the number of colleagues that would adhere to the recommendations, which used a 7 point Likert-scale (‘all colleagues’, ‘almost all colleagues’, ‘a majority’, ‘half’, ‘a minority’, ‘almost no colleague’, ‘not a single colleague’). This item does not concern clinicians’ own application of the recommendation, but rather their perspective on its broader applicability among colleagues. For this reason, this item will be reported separately in the text. Additionally, 16 self- administrated questions were added to the questionnaire to assess clinicians’ experience with the applicability of the recommendations (e.g. ‘to what extent were you able to have discussions with patients with incurable cancer on lifegoals, choices and which care suits them now and in the future?’). The response format was a 5-point Likert scale (‘very successful’, ‘successful’, ‘neutral’, ‘not successful’, ‘not successful at all’). The participating clinicians represented a range of specialties and disciplines involved in the care of patients with incurable cancer. It is possible that these clinicians had more affinity with palliative care than average; however, their diversity in background enhances the representativeness of the sample.

#### Patients’ experiences with the recommendations

To assess whether patients had received care in accordance with the recommendations, in month 3 participating clinicians asked all consecutive patients with incurable cancer who were admitted to the hospital or who visited the outpatient department to complete the questionnaire. Patients were eligible for inclusion if they were diagnosed with incurable cancer, aged 18 years or older and able to read in Dutch. Patients were identified with having incurable cancer based on clinical judgement. Patients received an envelope containing the questionnaire, an information letter and an informed consent form that they could complete at home. Questionnaires could be returned using the included return envelopes. The questionnaire included 27 self-administrated questions relating the different aspects of the recommendations (e.g. ‘Did you discuss your goals, choices and what care suits them now and in the future with a clinician?’). Patients were also asked about their experiences with the received care; whether they experienced the care as pleasant and helpful and if they were content with the care they received. These questions were answered on a 5-point Likert-scale ranging from ‘not at all’ to ‘very much’. Additionally the questionnaire included the European Organisation for Research and Treatment of Cancer In-patient Satisfaction with Care Questionnaire (EORTC IN-PATSAT32), which is a validated instrument measuring in-patient satisfaction with care [27]. Respondents assessed the skills of clinicians on a 5-point Likert scale (ranging from ‘poor’ to ‘excellent’). Scores were linearly recoded to a 0–100 scale with higher scores indicating higher satisfaction. The questionnaire was developed together with a patient representative of the Dutch Federation of Cancer Patients' Organisations.

### Data analysis

Descriptive statistics were used to describe the sociodemographic characteristics, the MIDI and self-administrated questions of the clinicians’ questionnaire, the EMR analysis, the experiences of patients, including their perceived satisfaction of care, continuity of care and continuity of information by patients. Based on previous research, aspects of the MIDI instrument were considered important for the applicability if ≥ 80 of the participants agreed or strongly agreed [28]. A priori, the minimum application of the intervention was set at 50%, meaning that at least 50% of the eligible patients should have received ACP, symptom burden assessment in all four dimensions and/or been offered SPCT consultation. If an ACP discussion had taken place, the outcomes should have been shared with the GP or involved institutional clinicians for at least 50% of these patients. Additionally, at least 50% of patients identified using the SQ 3 months should have been offered a consultation with the SPCT. The 50% threshold was chosen based on existing data, indicating that ACP, routine symptom monitoring and the SPCT are not yet common practice in Dutch hospitals [22, 29–31]. Given these low baseline levels of implementation as well as the relatively short duration of the pilot, a 50% implementation rate was considered a realistic and meaningful benchmark for assessing feasibility. A T-test for independent samples was used to compare satisfaction of care, continuity of care and continuity of information of patients who received care in accordance with the recommendations and those who did not. All analysis were conducted using Stata (version 17.0).

### Ethical considerations

The study was assessed by the Medical Ethical Committee of Brabant (METC NW2023-44) and exempt from full approval of an ethics committee.

## Results

### The use of the recommendations

In total, 542 EMRs of patients with incurable cancer were analysed. Of these, 251 (46%) included male patients. The median age of the patients was 69 years (ranging from 24–96). The three most common tumour types were lung (23%), breast (20%) and prostate (13%). 508 (94%) were records of outpatients and 34 (6%) of inpatients. All aspects of the recommendations reached the threshold of 50%, except for conducting ACP discussions (49%) (Table 1). Among patients expected to be in their last year of life (n = 220), the symptom burden on four dimensions and treatment decisions were recorded in 58% (n = 127). More than half of patients with an expected life expectancy of ≤ 3 months (n = 65) were offered a consultation with the SPCT (58%, n = 38).

**Table 1:** Applying the recommendations in daily practice: EMR analysis of patients with incurable cancer (N = 542)

	Yes % (n)
*Advance care planning*	
Was an ACP discussion conducted in the previous year?	49 (267)
If ACP was conducted: Were the outcomes of the ACP discussion shared with the GP and/or involved institutional physician?	84 (224)
*Routine symptom monitoring*	
A negative answer to the surprise question 12 months (‘Would I be surprised if this patient died in the next 12 months?’)	n = 220
If SQ 12 months is negative: Were the outcomes of symptom monitoring on the four dimensions and corresponding treatment decisions recorded?	58 (127)
*Involvement of SPCT*	
A negative answer to the surprise question 3 months (‘Would I be surprised if this patient died in the next 3 months?’)	n = 65
If SQ 3 negative: Were these patients offered a consultation with the SPCT?	58 (38)

### Applicability of recommendations from clinicians’ perspective

A total of 27 clinicians from three hospitals completed the questionnaire, ranging from 5–16 clinicians per hospital. One hospital did not return any questionnaires. Respondents were physicians (n = 13), nurses (n = 13), and nurse practitioner (n = 1) with a mean age of 46 years (SD 10). Most were female (n = 23) and more than half (n = 17) had received additional training in palliative care (Supplementary Table 2).

Between 21 and 23 clinicians agreed with the statements that the three recommendations were important to achieve timely integration palliative care (84–85%) (Table 2). A total of 22 clinicians (88%) agreed that the recommendation fitted in with their tasks. 20 clinicians (80%) stated they had the required knowledge for implementing it and that it aided in achieving timely integration of palliative care. Almost half of all clinicians thought that ‘a majority of their colleagues’, ‘almost all of their colleagues’ or ‘all of their colleagues’ would adhere to the recommendations of offering ACP (n = 13 (48%)), start routine symptom monitoring (n = 12 (46%)) and considering involving the SCPT in case of complexity and/or at patients’ request and offering a consultation in case of a life expectancy of < 3 months (n = 14 (56%)).

**Table 2:** Applicability according to clinicians: results of the MIDI instrument

	Recommendation on advance care planning^a^ (n = 27)	Recommendation on routine symptom monitoring^b^ (n = 26)	Recommendation on SPCT^c^ (n = 25)
	Agree % (n)	Agree % (n)	Agree % (n)
It is important for achieving timely integration of palliative care for my patients	**85 (23)**	**84 (21)**^d^	**84 (21)**
It fits in with the tasks for which I feel responsible when doing my work	78 (21)	73 (19)	**88 (22)**
Colleagues are supportive when implementing it	74 (20)	77 (20)	64 (16)
I am aware of its content	73 (19)^d^	68 (17)^d^	76 (19)
Patients will be satisfied	70 (19)	65 (17)	72 (18)
Patients will cooperate	70 (19)	69 (18)	72 (18)
I have the required knowledge to implement it	70 (19)	69 (18)	**80 (20)**
It will probably aid in achieving timely integration of palliative care for my patients	63 (17)	50 (13)	**80 (20)**
I feel able to implement the activities involved with it	59 (16)	48 (12)^d^	76 (19)
It benefits me	56 (15)	38 (10)	64 (16)

Bold indicates a score above the threshold of 80%

^a^Recommendation on ACP: Offer advance care planning (ACP) to all patient with incurable cancer and conduct a (follow-up) ACP discussion in the event of increased symptom burden, a life expectancy of < 1 year (identified using the Surprise Question (SQ) ‘Would I be surprised if this patient dies within 12 months?’) and/or a change in line of treatment. Record the outcomes of these discussions in the EMR and share them with the primary care physician or involved institutional physician

^b^Recommendation on routine symptom monitoring: Routine symptom monitoring and symptom management across the four dimensions of Quality of Life (QoL) (physical, psychological, social and spiritual) in patients with cancer and a life expectancy of < 1 year. Record the outcomes of symptom monitoring and corresponding treatment decisions in the EMR

^c^Recommendation on the SPCT: 3) Consider involving the SPCT in cases of complexity, such as symptom burden on multiple dimensions, and/or if the patient requests their involvement. Offer a consultation with the SPCT to patients with cancer and a life expectancy of < 3 months (identified using the adapted Surprise Question ‘Would I be surprised if this patient dies within 3 months?’)

^d^Missing n = 1

Most clinicians reported that they were able to have ACP discussions (ranging from n = 17 (63%) to n = 21 (78%)) (Table 3). Only 9 (33%) clinicians reported they were able to share the content with the GP or involved other institutional clinicians. Regarding symptom monitoring, 17 (63%) clinicians stated they were able to identify patients using the SQ, but only a minority monitored symptoms across the four dimensions (n = 11 (42%)) and recorded the outcomes and corresponding treatment decisions in the EMR (n = 5 (19%)). Most clinicians considered involving the SPCT in case of complexity and/or at patients’ request and offered a consultation in case of a life expectancy of < 3 months (ranging from n = 17 (65%) to n = 22 (85%)).

**Table 3:** Applicability according to clinicians: additional self-questions

To what extent were you able to…	Able % (n)	Neutral % (n)	Not able % (n)
*Recommendation on advance care planning (N* = *27)*			
…identify patients with incurable cancer^f^	96 (25)		4 (1)
…have an ACP discussion (again) when there is a life expectancy of < 1 year	78 (21)	19 (5)	4 (1)
…record the content of ACP discussions in the EMR	67 (18)	26 (7)	7 (2)
…have ACP discussions with patients with incurable cancer^f^	65 (17)	27 (7)	8 (2)
…have this discussion (again) when there is a change in line of therapy	63 (17)	26 (7)	11 (3)
…have this discussion (again) when there is an increased symptom burden?	63 (17)	22 (6)	15 (4)
…integrate these discussions into your daily clinical work	59 (16)	26 (7)	15 (4)
…share the content of these discussions with the GP or involved institutional physician?	33 (9)	37 (10)	30 (8)
*Recommendation on routine symptom monitoring (N* = *27)*			
…identify patients with incurable cancer and a life expectancy of < 1 year using the Surprise Question?	63 (17)	22 (6)	15 (4)
…pay attention to symptom monitoring and symptom treatment on the four dimensions (physical, psychological, social and spiritual)?^f^	42 (11)	42 (11)	15 (4)
…record outcomes of symptom monitoring on the four dimensions and corresponding treatment decisions in the electronic medical record?	19 (5)	44 (12)	37 (10)
*Recommendation on SPCT (N* = *27)*			
…consider involving the SCPT at the patients’ request?^f^	85 (22)	15 (4)	
…consider involving the SPCT in case of complexity, such as symptom burden on multiple dimensions?^f^	73 (19)	27 (7)	
…offer patients with an expected life expectancy of < 3 months a consultation with the SPCT?^g^	68 (17)	32 (8)	
…identify patients with incurable cancer and a life expectancy of < 3 months using the Surprise Question?^f^	65 (17)	27 (7)	8 (2)

^f^Missing n = 1

^g^Missing n = 2

### Patients’ experiences with the recommendations

A total of 70 patients with incurable cancer completed the questionnaire (Table 4). The mean age was 63 years, and most patients had prostate cancer (19%) or breast cancer (16%).

**Table 4:** Social demographic characteristics of patients (N = 70)

	% (n)
*Gender*	
Male	64 (45)
Female	36 (25)
Age (mean (SD); range min–max)	67 (12); 24–85
*Marital status*	
In a relationship, married and living together	74 (52)
In a relationship, not married and living together	9 (6)
In a relationship, not living together	3 (2)
Widow/widower/partner deceased	3 (2)
Single/no relationship	12 (8)
*Educational level*	
No education or primary education	3 (2)
Secondary (vocational) education	66 (46)
Bachelor’s, master’s or doctorate	29 (20)
*Other*	
M	3 (2)
*Tumour type*	
Colorectal	9 (6)
Breast	16 (11)
Prostate	19 (13)
Lung	11 (8)
Other^h^	36 (25)
m	10 (7)
*Setting*	
Outpatient	47 (33)
Inpatient	53 (37)

^h^Other tumours included melanoma, leukaemia, ovarium, brain, bladder, multiple myeloma, pancreas, gallbladder, bile duct, liver, lymph nodes and oesophagus)

In total, 62% of the patients reported to have had an ACP discussion. Whether symptoms had been monitored, according to patients, varied among the dimensions, ranging from 91% for the physical dimension to 38% for the spiritual dimension. More than half of the patients (53%) reported having had a consultation with a palliative care consultant (Table 5). Most patients found having an ACP discussion pleasant (70%) and helpful (74%). Regarding symptom monitoring, this varied per domain, with 70% finding it pleasant that the physician asked about physical symptoms and 46% regarding spiritual issues. When having a consultation with a member of the SPCT, 58% experienced it as pleasant and 42% as helpful. General satisfaction with care and continuity with care did not differ significantly between patients who did receive care according to the recommendations, except for symptom monitoring on the social dimension, respectively 83.1 (SD 18.7) vs 71.2 (SD 20.2), p = 0.03 and 88.2 (SD 16.2) vs 77.5 (SD 22.3), p = 0.03.

**Table 5:** Applicability according to patients, their experience of the received care and experienced satisfaction with care (N = 70)

**Advance care planning (n = 69)**^**i**^	**Yes % (n)**	**No % (n)**	
Did you discuss your goals, choices and what care suits them now and in the future with a clinician?	62 (43)	38 (26)	
I found it pleasant	70 (30)		
I found it helpful	74 (32)		
I am content with it	74 (32)		
	**Mean (SD)**	**Mean (SD)**	**P-value**
General satisfaction with care	77 (21)	75 (21)	0.66
Continuity of care	82 (22)	81 (19)	0.79
Continuity of information	89 (20)	90 (23)	0.91
**Routine symptom monitoring– physical dimension (n = 69)**^**i**^	**Yes % (n)**	**No % (n)**	
Did a clinician ask about your physical problems?	91 (63)	9 (6)	
I found it pleasant	70 (44)		
I found it helpful	67 (42)		
I am content with it	76 (48)		
	**Mean (SD)**	**Mean (SD)**	**P-value**
General satisfaction with care	77 (21)	71 (25)	0.50
Continuity of care	82 (21)	83 (18)	0.88
Continuity of information	90 (18)	78 (40)	0.16
**Routine symptom monitoring– psychological/emotional dimension (n = 70)**	**Yes % (n)**	**No % (n)**	
Did a clinician ask about your psychological/emotional problems?	57 (40)	43 (30)	
I found it pleasant	60 (25)		
I found it helpful	55 (23)		
I am content with it	64 (27)		
	**Mean (SD)**	**Mean (SD)**	**P-value**
General satisfaction with care	79 (19)	73 (23)	0.28
Continuity of care	85 (21)	78 (20)	0.16
Continuity of information	93 (15)	84 (26)	0.08
**Routine symptom monitoring– social dimension (n = 68)**^**j**^	**Yes % (n)**	**No % (n)**	
Did a clinician ask about your social problems?	46 (31)	54 (37)	
I found it pleasant	65 (20)		
I found it helpful	58 (18)		
I am content with it	68 (21)		
	**Mean (SD)**	**Mean (SD)**	**P-value**
General satisfaction with care	83 (19)	72 (20)	0.03*
Continuity of care	88 (16)	77 (22)	0.03*
Continuity of information	92 (17)	86 (24)	0.25
**Routine symptom monitoring– spiritual dimension (n = 69)**^**i**^	**Yes % (n)**	**No % (n)**	
Did a clinician ask about your spiritual challenges?	38 (26)	62 (43)	
I found it pleasant	46 (12)		
I found it helpful	50 (13)		
I am content with it	58 (15)		
	**Mean (SD)**	**Mean (SD)**	**P-value**
General satisfaction with care	80 (19)	74 (22)	0.30
Continuity of care	78 (25)	84 (18)	0.29
Continuity of information	90 (18)	89 (23)	0.91
**Specialist Palliative Care Team– informed about consultation (n = 68)**^**j**^	**Yes % (n)**	**No % (n)**	
Were you informed about the possibility of having a consultation with a PC consultant?	47 (32)	53 (36)	
I found it pleasant	39 (13)		
I found it helpful	36 (12)		
I am content with it	56 (18)		
	**Mean (SD)**	**Mean (SD)**	**P-value**
General satisfaction with care	77 (20)	76 (22)	0.97
Continuity of care	81 (22)	81 (20)	0.96
Continuity of information	92 (17)	87 (24)	0.37
**Specialist Palliative Care Team– having a consultation (n = 36)**^**k**^	**Yes % (n)**	**No % (n)**	
Did you have a consultation with a palliative care consultant?	53 (19)	47 (17)	
I found it pleasant	58 (11)		
I found it helpful	42 (8)		
I am content with it	53 (10)		
	**Mean (SD)**	**Mean (SD)**	**P-value**
General satisfaction with care	72 (20)	81 (24)	0.26
Continuity of care	79 (17)	90 (20)	0.07
Continuity of information	88 (19.9)	92 (25)	0.56

^i^Missing n = 1

^j^Missing n = 2

^k^Missing n = 34

## Discussion

### Main findings

This pilot study has assessed the clinical feasibility of implementing three recommendations for timely integration of palliative care in oncology. Clinicians were able to apply the recommendations in approximately half to two-thirds of their patients with incurable cancer. Moreover, clinicians rated all three recommendations as important, but some practical implications of the recommendations require further attention. Most patients for whom the recommendations were applied found this helpful and were satisfied with their care. Overall satisfaction with their care and continuity of care did not differ significantly between patients who received care according to the recommendations and those who did not, with the exception of symptom monitoring of the social dimension.

### Advance care planning

In the present study, clinicians were willing to integrate ACP discussions for patients with incurable cancer into their daily practice, however applying the ACP recommendation in clinical practice proved challenging. The recommendation on ACP scored just below the 50% threshold and a significant number of clinicians reported being ‘neutral’ or ‘not able’ to integrate ACP into their daily clinical practice. While this pilot study did not collect data on barriers experienced, there is a substantial body of literature on barriers for the integration of ACP. Barriers include knowledge (e.g. having insufficient understanding of ACP), environmental context and resources (e.g. time constraints), emotions (e.g. fear of diminishing patients’ hope) and skills (e.g. lack of training) [32]. To support clinicians and increase their knowledge and skills about ACP, a variety of tools are available to introduce and facilitate ACP discussions [33], both patient-facing tools [34] and interactive web-based ACP support tools [35].

While time is a well-known barrier, a recent randomised clinical trial demonstrated the durability of long-term ACP, with a reduction in the use of acute, complex or invasive care and an increase in palliative- and hospice care in a timely manner in patients’ disease trajectory [36]. This highlights the fact that the time investment required to integrate ACP will ultimately result in important benefits for clinicians, patients and the wider healthcare system.

Only 33% of clinicians shared the outcome of ACP discussions with the GP or other involved institutional clinicians, yet this coordination and collaboration with primary care providers is essential for proper integration of ACP. This finding is consistent with another national study that also showed suboptimal sharing of important information between oncologists and GPs [37]. A standardised format and transmural documentation is needed to improve this exchange and thus continuity of care. Preferably an automated report derived from the EMR that can be sent to all clinicians involved after an ACP discussion is conducted. Having a method of documentation that is accessible to different clinicians allows the ACP discussions to be conducted by different clinicians at different points in the disease trajectory, such as the treating physician, trained case managers, oncology nurses, or the GP. This reinforces the idea that ACP should be seen as an ongoing standard of care within the network of involved clinicians, rather than an optional consultation.

### Routine symptom monitoring

Integrating routine symptom monitoring into cancer care has been shown to improve health-related quality of life [38, 39]. Additionally, self-reported information can facilitate and improve the communication and discussion between clinicians and patients [40, 41]. Accordingly, the clinicians participating in the current study reported that they considered routine symptom monitoring to be important. However, clinicians were ambiguous regarding their ability to monitor symptom burden across the four dimensions and record the outcomes in the EMR. There are different ways in which clinicians monitor symptoms, including eliciting symptoms during clinical visits, often without a structured discussion format, to using a symptom diary that patients complete in a structural way. Proper integration of these methods requires a dedicated place in the EMR. And while a variety of digital methods and tools for monitoring symptoms with electronic patient-reported outcomes have been developed [42–45], many hospitals are still developing fitting (digital) infrastructure and standard operation of procedure into their clinical workflow.

Furthermore, less than half of the clinicians believed that their colleagues would adhere to the recommendation. This assumption may be due to known barriers to implementing symptom monitoring, such as concerns about additional workload, scepticism about the benefits for oncological treatment and usability issues [46, 47]. To use patient-reported outcomes, the system used must be applicable to all patients, easily adaptable, and compatible with the existing workflows [47, 48]. This finding may also be prompted by the fact that most clinicians who completed the post-assessment were additionally trained in palliative care and may therefore have had less positive experiences and perceptions of their generalist palliative care colleagues' motivation and ability to integrate palliative care in a timely manner.

Patients experienced symptom monitoring on the psychological, social and spiritual dimensions less comfortable and helpful than symptom monitoring on the physical dimension. This may also be related to the tendency of healthcare providers to focus only on the physical dimension (e.g., dyspnoea or pain) rather than adequately addressing psychological or social issues. Another possible explanation for patients’ reluctance to discuss psychosocial issues might be the stigma associated with psychosocial needs [49–52]. Additionally, a systematic review showed that patients vary widely in their appreciation of being asked about spirituality. Most of the included studies reported that patients found it appropriate for their physicians to ask about religion or spirituality. However, when dealing with spiritual issues, patients do not expect or want spiritual guidance from their physicians, but would rather talk to clergy [53].

### Specialist palliative care team

Most SPCTs in the Dutch hospitals have limited occupancy and therefore limited capacity. It is therefore not feasible to refer all patients with palliative care needs to the SPCT. It has therefore been suggested that the only sustainable model is a generalist-specialist model in which all clinicians provide basic or primary palliative care with the SPCT assisting with complex cases [54, 55]. However, it is known that the SPCT is often involved (too) late in the disease trajectory of patients with incurable cancer [31, 56].

This recommendation follows the results of a previous Delphi study in which clinicians, patients and relatives reached a consensus on the *minimum* thresholds for involving the SPCT in a generalist-specialist model [22]. However, if the SPCT is to be involved in cases of complexity, further research is needed to define complexity and identify ways of integrating the classification system into clinical practice [57]. In addition, timeliness may not be ensured if SPCT involvement is considered only for symptom burden on more than one dimension, as symptom burden increases significantly in the last phase of life, with the greatest increase in the last three months [58]. Appropriate symptom management at the end of life can be provided by palliative care generalists if they are skilled enough to provide high quality generalist palliative care. Elements of high-quality generalist palliative care include: general and psychosocial symptom assessment and management, providing patients with basic spiritual support, such as a framework to consider their goals and hopes and timely referral to a spiritual counsellor if needed; and starting the process of advance care planning (ACP) soon after the initial diagnosis of advanced cancer [59]. At present, however, palliative care training is not integrated into medical education and clinicians are not required to undertake additional training, although steps are being taken to improve the curriculum [60].

Not all patients in our pilot found it particularly helpful to be informed about the possibility to come into contact with palliative care consultants of the SPCT. This may be related to the misconceptions about palliative care, for example that it is the same as terminal care or hospice care, or that it means stopping all other treatments [61, 62].

### Timely palliative care

A substantial number of studies are dedicated to early palliative care. In these studies, early refers to the moment of diagnosis, prognosis or a combination of the two [6, 7, 10, 13, 15]. Specific triggers or characteristics as defined in the recommendations ensures that palliative care is not integrated too late. However, palliative care is a dynamic process and should be centred around patients’ needs [63]. Timely provision of palliative care therefore focusses on patients’ needs and implies that palliative care is delivered at the optimal moment for each patient [64]. The recommendations promote timely discussions about care wishes, a timely initiation of symptom monitoring across all four dimensions, ensuring a timely identification of patients’ needs. In the Dutch generalist-specialist model, palliative care generalists can attend to most needs, When necessary they can consult a consultant of the SPCT.

### Strengths and limitations

This study demonstrates the feasibility of three recommendations for the timely integration of palliative care into standard hospital oncology care. Our study has several strengths and limitations. A strength was that it followed the results of a Delphi study and the recommendations were therefore in line with current clinical practice and ambitions. Additionally, various methods were used to provide complimentary insights into the feasibility of the recommendations. Some limitations should also be noted. First, the clinicians using the recommendations in the pilot hospitals may have been more aware of the benefits of integrating palliative care into oncology practice. Therefore, both clinician and patient outcomes may have been more positive than in other hospitals. Second, when analysing the EMRs, the presence of an indication that an ACP discussion took place does not provide a detailed insight into the actual content or depth of the discussion. This also holds for the indication of an exchange of information with primary care physicians in the EMR, which does not indicate the method of exchange (via a telephone call or via a standard information letter). Third, as mentioned in the methods section, there may have been patients who had had ACP discussions before the start of the pilot and for whom there was no indication to have another ACP discussion during the pilot. Therefore, in the EMR analysis, we looked at whether patients had had an ACP discussion in the year prior to the pilot study, and if so, this would suggest that the recommendation was already being applied. However, the study did not collect data on when the ACP discussions took place. Fourth, the EMR analysis was not always performed by the treating physician, and the investigator was asked to answer the surprise questions of one year and three months based on his or her own clinical judgement. Fifth, the clinician and patient questionnaires included self-administered questions that were not formally pre-tested. While they were developed in close collaboration with clinical experts, their reliability and validity have not been established, which may affect the generalizability of the findings. Sixth, it is important to acknowledge the possibility of selection bias in the patient sample, as participation was limited to those who chose to complete and return the questionnaire—a common limitation in questionnaire-based studies. Finally, patient assessment was mainly explorative and descriptive. The pilot was of short duration, which made it impossible to measure real effects over time.

## Conclusion

Implementing the three recommendations regarding ACP, routine symptom monitoring, and involvement of the Specialist Palliative Care Team (SPCT) in daily oncological care for patients with advanced cancer seems feasible, as clinicians were able to apply the recommendations in almost half of the patients and found them to be important. To truly improve care with the use of these recommendations some practical issues need to be addressed regarding continuity of care, including information exchange about ACP and symptom management amongst all involved clinicians within the hospital and with primary care clinicians.

## Electronic supplementary material

Below is the link to the electronic supplementary material.

Supplementary Material 1

## Data Availability

The datasets generated and/or analysed during the current study are not publicly available but are available from the corresponding author on reasonable request.

## Abbreviations

ACP: Advance care planning
EMR: Electronic medical record
MIDI: Measurement Instrument for Determinants of Innovations
SPCT: Specialist Palliative Care Team
WHO: World Health Organisation

## Footnotes

1 Advance care planning enables individuals to define goals and preferences for future medical treatment and care, to discuss these goals and preferences with family and clinicians, and to record and review these preferences if appropriate. [24]

## References

[1] Geijteman EC, Kuip EJ, Oskam J, Lees D, Bruera E. Illness trajectories of incurable solid cancers. BMJ. 2024;384:e076625. 38428972 10.1136/bmj-2023-076625 PMC10905388

[2] Bray F, Laversanne M, Sung H, Ferlay J, Siegel RL, Soerjomataram I, et al. Global cancer statistics 2022: GLOBOCAN estimates of incidence and mortality worldwide for 36 cancers in 185 countries. CA Cancer J Clin. 2024;74(3):229–63. 38572751 10.3322/caac.21834

[3] Etkind SN, Bone AE, Gomes B, Lovell N, Evans CJ, Higginson IJ, et al. How many people will need palliative care in 2040? Past trends, future projections and implications for services. BMC Med. 2017;15(1):102. 28514961 10.1186/s12916-017-0860-2 PMC5436458

[4] Sepúlveda C, Marlin A, Yoshida T, Ullrich A. Palliative care: the World Health Organization’s global perspective. J Pain Symptom Manag. 2002;24(2):91–6. 10.1016/s0885-3924(02)00440-2 12231124

[5] Temel JS, Greer JA, Muzikansky A, Gallagher ER, Admane S, Jackson VA, et al. Early palliative care for patients with metastatic non–small-cell lung cancer. N Engl J Med. 2010;363(8):733–42. 20818875 10.1056/NEJMoa1000678

[6] Maltoni M, Scarpi E, Dall’Agata M, Schiavon S, Biasini C, Codeca C, et al. Systematic versus on-demand early palliative care: a randomised clinical trial assessing quality of care and treatment aggressiveness near the end of life. Eur J Cancer. 2016;69:110–8. 27821313 10.1016/j.ejca.2016.10.004

[7] Vanbutsele G, Pardon K, Van Belle S, Surmont V, De Laat M, Colman R, et al. Effect of early and systematic integration of palliative care in patients with advanced cancer: a randomised controlled trial. Lancet Oncol. 2018;19(3):394–404. 29402701 10.1016/S1470-2045(18)30060-3

[8] Vanbutsele G, Van Belle S, Surmont V, De Laat M, Colman R, Eecloo K, et al. The effect of early and systematic integration of palliative care in oncology on quality of life and health care use near the end of life: a randomised controlled trial. Eur J Cancer. 2020;124:186–93. 31812934 10.1016/j.ejca.2019.11.009

[9] Fulton JJ, LeBlanc TW, Cutson TM, Porter Starr KN, Kamal A, Ramos K, et al. Integrated outpatient palliative care for patients with advanced cancer: a systematic review and meta-analysis. Palliat Med. 2019;33(2):123–34. 30488781 10.1177/0269216318812633 PMC7069657

[10] Temel JS, Greer JA, El-Jawahri A, Pirl WF, Park ER, Jackson VA, et al. Effects of early integrated palliative care in patients with lung and GI cancer: a randomized clinical trial. J Clin Oncol. 2017;35(8):834–41. 28029308 10.1200/JCO.2016.70.5046 PMC5455686

[11] Bakitas M, Lyons KD, Hegel MT, Balan S, Brokaw FC, Seville J, et al. Effects of a palliative care intervention on clinical outcomes in patients with advanced cancer: the Project ENABLE II randomized controlled trial. JAMA. 2009;302(7):741–9. 19690306 10.1001/jama.2009.1198 PMC3657724

[12] El-Jawahri A, Greer JA, Pirl WF, Park ER, Jackson VA, Back AL, et al. Effects of early integrated palliative care on caregivers of patients with lung and gastrointestinal cancer: a randomized clinical trial. Oncologist. 2017;22(12):1528. 28894017 10.1634/theoncologist.2017-0227 PMC5728034

[13] Zimmermann C, Swami N, Krzyzanowska M, Hannon B, Leighl N, Oza A, et al. Early palliative care for patients with advanced cancer: a cluster-randomised controlled trial. Lancet. 2014;383(9930):1721–30. 24559581 10.1016/S0140-6736(13)62416-2

[14] Boddaert MS, Pereira C, Adema J, Vissers KCP, van der Linden YM, Raijmakers NJH, et al. Inappropriate end-of-life cancer care in a generalist and specialist palliative care model: a nationwide retrospective population-based observational study. BMJ Support Palliat Care. 2020;12(e1):e137–45. 10.1136/bmjspcare-2020-002302 PMC9120402 33355176

[15] Bakitas MA, Tosteson TD, Li Z, Lyons KD, Hull JG, Li Z, et al. Early versus delayed initiation of concurrent palliative oncology care: patient outcomes in the ENABLE III randomized controlled trial. J Clin Oncol. 2015;33(13):1438–45. 25800768 10.1200/JCO.2014.58.6362 PMC4404422

[16] Haun MW, Estel S, Rucker G, Friederich HC, Villalobos M, Thomas M, et al. Early palliative care for adults with advanced cancer. Cochrane Database Syst Rev. 2017;6:CD011129. 28603881 10.1002/14651858.CD011129.pub2 PMC6481832

[17] Jordan K, Aapro M, Kaasa S, Ripamonti C, Scotté F, Strasser F, et al. European Society for Medical Oncology (ESMO) position paper on supportive and palliative care. Ann Oncol. 2018;29(1):36–43. 29253069 10.1093/annonc/mdx757

[18] Hui D, Cherny NI, Wu J, Liu D, Latino NJ, Strasser F. Indicators of integration at ESMO Designated Centres of Integrated Oncology and Palliative Care. ESMO Open. 2018;3(5):e000372. 30018816 10.1136/esmoopen-2018-000372 PMC6045723

[19] Ferrell BR, Temel JS, Temin S, Alesi ER, Balboni TA, Basch EM, et al. Integration of palliative care into standard oncology care: American Society of Clinical Oncology clinical practice guideline update. J Clin Oncol. 2017;35(1):96–112. 28034065 10.1200/JCO.2016.70.1474

[20] Kaasa S, Loge JH, Aapro M, Albreht T, Anderson R, Bruera E, et al. Integration of oncology and palliative care: a Lancet Oncology Commission. Lancet Oncol. 2018;19(11):e588–653. 30344075 10.1016/S1470-2045(18)30415-7

[21] Hui D, Bansal S, Strasser F, Morita T, Caraceni A, Davis M, et al. Indicators of integration of oncology and palliative care programs: an international consensus. Ann Oncol. 2015;26(9):1953–9. 26088196 10.1093/annonc/mdv269 PMC4551157

[22] Heipon CS, Brom L, van der Linden YM, Tange D, Reyners AK, Raijmakers NJ. Characteristics of timely integration of palliative care into oncology hospital care for patients with incurable cancer: results of a Delphi Study. Support Care Cancer. 2024;32(5):324. 38700723 10.1007/s00520-024-08508-0

[23] Fleuren MA, Paulussen TG, Van Dommelen P, Van Buuren S. Towards a measurement instrument for determinants of innovations. Int J Qual Health Care. 2014;26(5):501–10. 24951511 10.1093/intqhc/mzu060 PMC4195468

[24] Rietjens JAC, Sudore RL, Connolly M, van Delden JJ, Drickamer MA, Droger M, et al. Definition and recommendations for advance care planning: an international consensus supported by the European Association for Palliative Care. Lancet Oncol. 2017;18(9):e543–51. 28884703 10.1016/S1470-2045(17)30582-X

[25] Sharma A, Minh Duc NT, Luu Lam Thang T, Nam NH, Ng SJ, Abbas KS, et al. A consensus-based checklist for reporting of survey studies (CROSS). J Gen Intern Med. 2021;36(10):3179–87. 33886027 10.1007/s11606-021-06737-1 PMC8481359

[26] Von Elm E, Altman DG, Egger M, Pocock SJ, Gøtzsche PC, Vandenbroucke JP. The Strengthening the Reporting of Observational Studies in Epidemiology (STROBE) statement: guidelines for reporting observational studies. Lancet. 2007;370(9596):1453–7. 18064739 10.1016/S0140-6736(07)61602-X

[27] Neijenhuijs KI, Jansen F, Aaronson NK, Brédart A, Groenvold M, Holzner B, et al. A systematic review of the measurement properties of the European Organisation for Research and Treatment of Cancer In-patient Satisfaction with Care Questionnaire, the EORTC IN-PATSAT32. Support Care Cancer. 2018;26:2551–60. 29732482 10.1007/s00520-018-4243-9 PMC6018571

[28] Verberne LM, Kars MC, Schepers SA, Schouten-van Meeteren AY, Grootenhuis MA, van Delden JJ. Barriers and facilitators to the implementation of a paediatric palliative care team. BMC Palliat Care. 2018;17:1–8. 10.1186/s12904-018-0274-8 PMC5810030 29433576

[29] Netherlands Comprehensive Cancer Center (IKNL). Palliatieve Zorg in Nederlandse ziekenhuizen: resultaten 2023. 2024.

[30] van Velzen N, Brom L, van der Vorst ML, Kiers M, Wagemans M, Kazimier H, et al. Development of specialist palliative care in Dutch hospitals between 2014 and 2020: a repeated survey. BMC Palliat Care. 2025;24(1):20. 39849515 10.1186/s12904-025-01657-x PMC11755811

[31] Boddaert MS, Stoppelenburg A, Hasselaar J, van der Linden YM, Vissers KCP, Raijmakers NJH, et al. Specialist palliative care teams and characteristics related to referral rate: a national cross-sectional survey among hospitals in the Netherlands. BMC Palliat Care. 2021;20(1):175. 34758792 10.1186/s12904-021-00875-3 PMC8582112

[32] Guccione L, Fullerton S, Gough K, Hyatt A, Tew M, Aranda S, et al. Why is advance care planning underused in oncology settings? A systematic overview of reviews to identify the benefits, barriers, enablers, and interventions to improve uptake. Front Oncol. 2023;13:1040589. 37188202 10.3389/fonc.2023.1040589 PMC10175822

[33] Myers J, Cosby R, Gzik D, Harle I, Harrold D, Incardona N, et al. Provider tools for advance care planning and goals of care discussions: a systematic review. Am J Hosp Palliat Med®. 2018;35(8):1123–32. 10.1177/1049909118760303 29529884

[34] Riley SR, Voisin C, Stevens EE, Bose-Brill S, Moss KO. Tools for tomorrow: a scoping review of patient-facing tools for advance care planning. Palliat Care Soc Pract. 2024;18:26323524241263108. 39045292 10.1177/26323524241263108 PMC11265253

[35] Dupont C, Smets T, Monnet F, Pivodic L, De Vleminck A, Van Audenhove C, et al. Publicly available, interactive web-based tools to support advance care planning: systematic review. J Med Internet Res. 2022;24(4):e33320. 35442207 10.2196/33320 PMC9069298

[36] Patel MI, Agrawal M, Blayney DW, Bundorf MK, Milstein A. Long-term engagement of patients with advanced cancer: results from the EPAC randomized clinical trial. JAMA Oncol. 2024;10:905–11. 38814627 10.1001/jamaoncol.2024.1221 PMC11140577

[37] Ermers DJ, van Bussel KJ, Perry M, Engels Y, Schers HJ. Advance care planning for patients with cancer in the palliative phase in Dutch general practices. Fam Pract. 2019;36(5):587–93. 30535044 10.1093/fampra/cmy124

[38] Basch E, Deal AM, Kris MG, Scher HI, Hudis CA, Sabbatini P, et al. Symptom monitoring with patient-reported outcomes during routine cancer treatment: a randomized controlled trial. J Clin Oncol. 2016;34(6):557–65. 26644527 10.1200/JCO.2015.63.0830 PMC4872028

[39] Billingy NE, Tromp VN, Aaronson NK, Hoek RJ, Bogaard HJ, Onwuteaka-Philipsen BD, et al. Quality of life after patient-initiated vs physician-initiated response to symptom monitoring: the SYMPRO-Lung trial. JNCI J Natl Cancer Inst. 2023;115(12):1515–25. 37603720 10.1093/jnci/djad159 PMC10699799

[40] Basch E, Stover AM, Schrag D, Chung A, Jansen J, Henson S, et al. Clinical utility and user perceptions of a digital system for electronic patient-reported symptom monitoring during routine cancer care: findings from the PRO-TECT trial. JCO Clin Cancer Inform. 2020;4:947–57. 33112661 10.1200/CCI.20.00081 PMC7768331

[41] Yang LY, Manhas DS, Howard AF, Olson R. Patient-reported outcome use in oncology: a systematic review of the impact on patient-clinician communication. Support Care Cancer. 2018;26:41–60. 28849277 10.1007/s00520-017-3865-7

[42] Absolom K, Warrington L, Hudson E, Hewison J, Morris C, Holch P, et al. Phase III randomized controlled trial of eRAPID: eHealth intervention during chemotherapy. J Clin Oncol. 2021;39(7):734–47. 33417506 10.1200/JCO.20.02015

[43] Schuit AS, Holtmaat K, Lissenberg-Witte BI, Eerenstein SE, Zijlstra JM, Eeltink C, et al. Efficacy of the eHealth application Oncokompas, facilitating incurably ill cancer patients to self-manage their palliative care needs: a randomized controlled trial. Lancet Reg Health Eur. 2022;18:100390. 35496496 10.1016/j.lanepe.2022.100390 PMC9046636

[44] Basch E, Schrag D, Jansen J, Henson S, Stover AM, Spears P, et al. Digital symptom monitoring with patient-reported outcomes in community oncology practices: a US national cluster randomized trial. J Clin Oncol. 2021;39:349527–349527.

[45] Lizée T, Basch E, Trémolières P, Voog E, Domont J, Peyraga G, et al. Cost-effectiveness of web-based patient-reported outcome surveillance in patients with lung cancer. J Thorac Oncol. 2019;14(6):1012–20. 30776447 10.1016/j.jtho.2019.02.005

[46] Kiderlen TR, Schnack A, de Wit M. Essential barriers and considerations for the implementation of electronic patient-reported outcome (ePRO) measures in oncological practice: contextualizing the results of a feasibility study with existing literature. J Public Health. 2023;31(12):2071–88. 10.1007/s10389-022-01767-3 PMC9613453 36320803

[47] Lopez CJ, Jones JM, Campbell KL, Bender JL, Strudwick G, Langelier DM, et al. A pre-implementation examination of barriers and facilitators of an electronic prospective surveillance model for cancer rehabilitation: a qualitative study. BMC Health Serv Res. 2024;24(1):17. 38178095 10.1186/s12913-023-10445-3 PMC10768357

[48] Minteer SA, Cheville A, Tesch N, Griffin JM, Austin JD, Mitchell S, et al. Implementing cancer symptom management interventions utilizing patient-reported outcomes: a pre-implementation evaluation of barriers and facilitators. Support Care Cancer. 2023;31(12):697. 37962699 10.1007/s00520-023-08114-6 PMC10645625

[49] Fernández-Feito A, Alonso-Iglesias C, Paz-Zulueta M, Pellico-López A. Exploring psychosocial needs of patients with cancers through the lens of the physicians and nurses: a qualitative study. J Nurs Manag. 2024;2024(1):2175517. 40224851 10.1155/2024/2175517 PMC11919211

[50] Holland JC, Kelly BJ, Weinberger MI. Why psychosocial care is difficult to integrate into routine cancer care: stigma is the elephant in the room. J Natl Compr Cancer Netw. 2010;8(4):362–6. 10.6004/jnccn.2010.0028 20410331

[51] Parmet T, Yusufov M, Braun IM, Pirl WF, Matlock DD, Sannes TS. Willingness toward psychosocial support during cancer treatment: a critical yet challenging construct in psychosocial care. Transl Behav Med. 2023;13(7):511–7. 36940406 10.1093/tbm/ibac121 PMC10465092

[52] Schuit AS, Holtmaat K, van Zwieten V, Aukema EJ, Gransier L, Cuijpers P, et al. Organizing psycho-oncological care for cancer patients: the patient’s perspective. Front Psychol. 2021;12:625117. 33967892 10.3389/fpsyg.2021.625117 PMC8100060

[53] Best M, Butow P, Olver I. Do patients want doctors to talk about spirituality? A systematic literature review. Patient Educ Couns. 2015;98(11):1320–8. 26032908 10.1016/j.pec.2015.04.017

[54] Periyakoil VS, Gunten CF, Fischer S, Pantilat S, Quill T. Generalist versus specialist palliative medicine. J Palliat Med. 2022;25(2):193–9. 35103529 10.1089/jpm.2021.0644 PMC9022124

[55] Quill TE, Abernethy AP. Generalist plus specialist palliative care—creating a more sustainable model. N Engl J Med. 2013;368(13):1173–5. 23465068 10.1056/NEJMp1215620

[56] Adamidis F, Baumgartner NS, Kitta A, Kum L, Ecker F, Bär J, et al. Timely integration of palliative care. The reality check. A retrospective analysis. Support Cancer. 2024;32(8):518. 10.1007/s00520-024-08721-x PMC11254969 39017732

[57] Grant M, de Graaf E, Teunissen S. A systematic review of classifications systems to determine complexity of patient care needs in palliative care. Palliat Med. 2021;35(4):636–50. 33706600 10.1177/0269216321996983 PMC8022082

[58] Versluis MA, Raijmakers NJ, Baars A, van den Beuken-van Everdingen MH, de Graeff A, Hendriks MP, et al. Trajectories of health-related quality of life and symptom burden in patients with advanced cancer towards the end of life: longitudinal results from the eQuiPe study. Cancer. 2024;130(4):609–17. 37831749 10.1002/cncr.35060

[59] Bickel KE, McNiff K, Buss MK, Kamal A, Lupu D, Abernethy AP, et al. Defining high-quality palliative care in oncology practice: an American Society of Clinical Oncology/American Academy of Hospice and Palliative Medicine guidance statement. J Oncol Pract. 2016;12(9):e828–38. 27531376 10.1200/JOP.2016.010686

[60] de Bruin J, Verhoef M-J, Slaets JP, van Bodegom D. End-of-life care in the Dutch medical curricula. Perspect Med Educ. 2018;7:325–31. 30187388 10.1007/s40037-018-0447-4 PMC6191393

[61] Flieger SP, Chui K, Koch-Weser S. Lack of awareness and common misconceptions about palliative care among adults: insights from a national survey. J Gen Intern Med. 2020;35:2059–64. 32157652 10.1007/s11606-020-05730-4 PMC7351936

[62] Parajuli J, Chen ZJ, Walsh A, Williams GR, Sun V, Bakitas M. Knowledge, beliefs, and misconceptions about palliative care among older adults with cancer. J Geriatr Oncol. 2023;14(1):101378. 36182659 10.1016/j.jgo.2022.09.007

[63] Sedhom R, Shulman LN, Parikh RB. Precision palliative care as a pragmatic solution for a care delivery problem. J Clin Oncol. 2023;41(16):2888. 37084327 10.1200/JCO.22.02532 PMC10414742

[64] Hui D, Heung Y, Bruera E. Timely palliative care: Personalizing the process of referral. Cancers. 2022;14(4):1047. 35205793 10.3390/cancers14041047 PMC8870673

