# Supplementary material for: Timely integration of palliative care into oncology in hospitals in the Netherlands: a feasibility study
Source: BMC Health Serv Res. 2025 Sep 30;25:1231. doi: 10.1186/s12913-025-13199-2 (PMC12481736; doi:10.1186/s12913-025-13199-2)
Supplement: Supplementary file 1 — Supplementary Material 1 [file 12913_2025_13199_MOESM1_ESM.docx]

**Supplements**

**Supplementary material 1 Questionnaire for clinicians assessing the applicability of the recommendations**

**Questionnaire on applying the provisional recommendations for the timely integration of palliative care**

In recent months, three provisional recommendations have been applied in your department to patients with incurable cancer. This questionnaire is about the applicability of these recommendations in practice. There are no right or wrong answers, it is about your personal opinion.

We would like to ask you to complete the enclosed questionnaire and look back on your experiences of working with the recommendations over **the past month.**

The questionnaire talks about ‘recommendations’, here you can read ‘preliminary recommendations’. The recommendations will be addressed one by one. Responses to the questionnaire will be kept confidential and used only anonymously.

Would you please complete the questionnaire within **2 weeks**? Thank you!

| Date   \|  \|  \| - \|  \|  \| - \|  \|  \|  \|  \| \| --- \| --- \| --- \| --- \| --- \| --- \| --- \| --- \| --- \| --- \|   Questionnairenumber   \|  \|  \|  \| \| --- \| --- \| --- \| |
| --- | --- | --- | --- | --- | --- | --- | --- | --- | --- | --- | --- | --- | --- |

**Instructions**

- Complete the questionnaire with a black or blue pen (not a felt-tip pen).
- Put a clear cross in the answer box.
- If you fill in a wrong answer, leave the wrong cross and colour the right box completely black.
- Tick one box for each question.

| **General questions** |
| --- |

| i. | What is your age? |
| --- | --- |

|  |  |
| --- | --- |

ii. What is your gender?

| ❒ | Male |
| --- | --- |
| ❒ | Female |
| ❒ | Other |

iii. What is your profession?

| ❒ | Physician |
| --- | --- |
| ❒ | Nurse practitioner |
| ❒ | Nurse |
| ❒ | Other, namely… |
|  |  |

iv. Are you additionally trained in palliative care?

| ❒ | Yes | |
| --- | --- | --- |
|  | Yes, namely… | |
| ❒ | 2-years palliative care continuing medical education for physicians | |
| ❒ | 9-day course in palliative care for physicians | |
| ❒ | Nurses with 1-year palliative care continuing nursing education | |
| ❒ | Courses on palliative care | |
| ❒ | Internship palliative care within the hospital | |
| ❒ | Other, namely…. | |
|  |  |  |

| **Applicability of the recommendation on advance care planning** | | | | | | |
| --- | --- | --- | --- | --- | --- | --- |
| These questions are about the preliminary recommendation on proactive care planning that you have applied in recent months. The questions concern your experience in applying this recommendation.  Please tick one box per line to indicate your answer: | | | | | | |
|  |  | Very successful | Successful | Neutral | Did not succeed | Did not succeed at all |
| 1. | To what extent have you succeeded in identifying patients with incurable cancer? | ❒ | ❒ | ❒ | ❒ | ❒ |
| 2. | To what extent did you succeed in having conversations with these identified patients about life goals, choices and which care fits them now and in the future? | ❒ | ❒ | ❒ | ❒ | ❒ |
| 3. | To what extent did you succeed in having this conversation (again) when the symptom burden of patients with incurable cancer increases? | ❒ | ❒ | ❒ | ❒ | ❒ |
|  |  |  |  |  |  |  |
|  |  | Very successful | Successful | Neutral | Did not succeed | Did not succeed at all |
|  |  |  |  |  |  |  |
| 4. | To what extent did you succeed in having this conversation (again) in patients with incurable cancer and a life expectancy of <1 year? | ❒ | ❒ | ❒ | ❒ | ❒ |
| 5. | To what extent were you successful in having this conversation (again) in patients with incurable cancer and a change in treatment trajectory? | ❒ | ❒ | ❒ | ❒ | ❒ |
| 6. | To what extent have you managed to integrate these conversations into your daily tasks? | ❒ | ❒ | ❒ | ❒ | ❒ |
|  |  |  |  |  |  |  |
|  |  | Very successful | Successful | Neutral | Did not succeed | Did not succeed at all |
| 7. | To what extent were you successful in recording the content of these conversations in the EMR? | ❒ | ❒ | ❒ | ❒ | ❒ |
| 8. | To what extent did you manage to share the content of these conversations with the general practitioner or institution doctors involved? | ❒ | ❒ | ❒ | ❒ | ❒ |

| **Determinants related to provisional recommendation** | | | | | | |
| --- | --- | --- | --- | --- | --- | --- |
| The questions below are questions of the Measurement Instrument for Determinants of Innovations (MIDI) (see: <https://www.tno.nl/media/6077/fleuren_et_al_midi_measurement_instrument.pdf>). They are about the procedural clarity, correctness, completeness, complexity, compatibility, observability and relevance for patients of the provisional recommendation on proactive care planning that you have applied in recent months.  Please tick one box per line to indicate your answer: | | | | | | |
|  |  | Completely disagree | Disagree | Neither disagree, nor agree | Agree | Completely agree |
| 9. | The recommendation clearly describes the activities I should perform and in which order. | ❒ | ❒ | ❒ | ❒ | ❒ |
| 10. | The recommendation is based on factually correct knowledge. | ❒ | ❒ | ❒ | ❒ | ❒ |
| 11. | The recommendation provides all the information and materials needed to work with it properly. | ❒ | ❒ | ❒ | ❒ | ❒ |
| 12. | The recommendation is too complex for me to use. | ❒ | ❒ | ❒ | ❒ | ❒ |
| 13. | The recommendation is a good match for how I am used to working. | ❒ | ❒ | ❒ | ❒ | ❒ |
| 14 | The outcomes of applying the recommendation are clearly observable. | ❒ | ❒ | ❒ | ❒ | ❒ |
| 15. | I think the recommendation is relevant for my clients. | ❒ | ❒ | ❒ | ❒ | ❒ |

| **Determinants associated with the user** | | | | | | | | | | | | | | |
| --- | --- | --- | --- | --- | --- | --- | --- | --- | --- | --- | --- | --- | --- | --- |
| The questions below are questions of the Measurement Instrument for Determinants of Innovations (MIDI) (see: <https://www.tno.nl/media/6077/fleuren_et_al_midi_measurement_instrument.pdf>). They are about the benefit to you as a healthcare provider and the outcomes for your patients when applying the provisional recommendation on proactive care planning. | | | | | | | | | | | | | | |
| Please tick one box per line to indicate your answer: | | | | | | | | | | | | | | |
|  | |  | | | Completely disagree | | Disagree | | Neither disagree, nor agree | | Agree | | Completely agree | |
| 16. | | Applying this recommendation in daily clinical practice benefits me. | | | ❒ | | ❒ | | ❒ | | ❒ | | ❒ | |
| 17a. | | I think it is important to achieve the following objectives for my patients: the timely integration of palliative care in oncology. | | | ❒ | | ❒ | | ❒ | | ❒ | | ❒ | |
|  | |  | | |  | |  | |  | |  | |  | |
|  | |  | | | Most definitely not | | Definitely not | | Maybe | | Definitely | | Most definitely | |
| 18b. | | I expect that applying the recommendation will actually achieve the following objectives for my patients: the timely integration of palliative care in oncology. | | | ❒ | | ❒ | | ❒ | | ❒ | | ❒ | |
|  | |  | | |  | |  | |  | |  | |  | |
|  | |  | | |  | |  | |  | |  | |  | |
|  | |  | | | Completely disagree | | Disagree | | Neither disagree, nor agree | | Agree | | Completely agree | |
| 19. | | I feel it is my responsibility as a professional to apply this recommendation. | | | ❒ | | ❒ | | ❒ | | ❒ | | ❒ | |
| 20. | | Patients will generally be satisfied if I apply this recommendation. | | | ❒ | | ❒ | | ❒ | | ❒ | | ❒ | |
| 21. | | Patients will generally cooperate if I apply this recommendation. | | | ❒ | | ❒ | | ❒ | | ❒ | | ❒ | |
| 22. | | I can count on adequate assistance from my colleagues if I need it to | | | ❒ | | ❒ | | ❒ | | ❒ | | ❒ | |
|  | |  | | |  | |  | |  | |  | |  | |
|  |  | | No colleagues | Almost no colleagues | | A minority | | Half of my colleagues | | A majority | | Almost all colleagues | | All colleagues |
| 23. | In your opinion, what proportion of the colleagues in your organisation for whom the recommendation is intended will actually apply the recommendation? | | ❒ | ❒ | | ❒ | | ❒ | | ❒ | | ❒ | | ❒ |
|  |  | |  |  | |  | |  | |  | |  | |  |
|  | |  | | | Most definitely not | | Definitely not | | Maybe | | Definitely | | Most definitely | |
| 24. | | Should you wish to do so, do you think you can apply the recommendation in your daily clinical practice? | | | ❒ | | ❒ | | ❒ | | ❒ | | ❒ | |
|  | |  | | |  | |  | |  | |  | |  | |
|  | |  | | | Completely disagree | | Disagree | | Neither disagree, nor agree | | Agree | | Completely agree | |
| 25. | | I know enough to apply this recommendation. | | | ❒ | | ❒ | | ❒ | | ❒ | | ❒ | |
|  | |  | | |  | |  | |  | |  | |  | |
| 26. | | To what extent are you informed about the content of the recommendation? | | | ❒ | | ❒ | | ❒ | | ❒ | | ❒ | |
|  | |  | | |  | |  | |  | |  | |  | |

| **Determinants associated with the organisation** | | | | | | |
| --- | --- | --- | --- | --- | --- | --- |
| The questions below are questions of the Measurement Instrument for Determinants of Innovations (MIDI) (see: <https://www.tno.nl/media/6077/fleuren_et_al_midi_measurement_instrument.pdf>). They are about your hospital's organisation in applying the recommendation on proactive care planning.  Please tick one box per line to indicate your answer: | | | | | | |
|  |  |  |  |  |  |  |
|  |  | Yes | No |  |  |  |
| 27. | Has the management set up formal arrangements in your hospital relating to applying this recommendation (in policy plans, work plans and so on)? | ❒ | ❒ |  |  |  |
|  |  |  |  |  |  |  |
|  |  | Completely disagree | Disagree | Neither disagree, nor agree | Agree | Completely agree |
| 28. | In my hospital, there are arrangements in place so that staff who apply the recommendation and leave the hospital are replaced in good time by employees who are/will be adequately prepared to take over | ❒ | ❒ | ❒ | ❒ | ❒ |
| 29. | There are enough people in our hospital to apply the recommendation as intended. | ❒ | ❒ | ❒ | ❒ | ❒ |
| 30. | There are enough financial resources available to apply the recommendation as intended. | ❒ | ❒ | ❒ | ❒ | ❒ |
| 31. | Our hospital provides me with enough time to apply the recommendation as intended in my day-to-day work. | ❒ | ❒ | ❒ | ❒ | ❒ |
|  |  |  |  |  |  |  |
|  |  | Completely disagree | Disagree | Neither disagree, nor agree | Agree | Completely agree |
|  |  |  |  |  |  |  |
| 32. | Our hospital provides me with enough materials and other resources or facilities necessary for applying the recommendation as intended | ❒ | ❒ | ❒ | ❒ | ❒ |
|  |  |  |  |  |  |  |
|  |  | Yes | No |  |  |  |
| 33. | In my hospital, one or more people have been designated to coordinate the process of applying the recommendation. | ❒ | ❒ |  |  |  |
|  |  |  |  |  |  |  |
| 34. | Are there, in addition to the implementation of the recommendation, any other changes in the hospital affecting the implementation of the recommendation now or in the foreseeable future (reorganisation, merger, cuts, staffing changes, other recommendations)? | ❒ | ❒ |  |  |  |
|  |  |  |  |  |  |  |
|  |  | Completely disagree | Disagree | Neither disagree, nor agree | Agree | Completely agree |
| 35. | It is easy for me to find information in my hospital about applying the recommendation as intended. | ❒ | ❒ | ❒ | ❒ | ❒ |
| 36. | In my hospital, feedback is regularly provided about progress with the implementation of the recommendation | ❒ | ❒ | ❒ | ❒ | ❒ |

| **Determinants associated with the socio-political context** | | | | | | |
| --- | --- | --- | --- | --- | --- | --- |
| The question below is a question of the Measurement Instrument for Determinants of Innovations (MIDI) (see: <https://www.tno.nl/media/6077/fleuren_et_al_midi_measurement_instrument.pdf>). It is about laws and regulations for the recommendation on proactive care planning.  Please tick one box per line to indicate your answer: | | | | | | |
| 37. | The activities listed in the recommendation fit in well with existing legislation and regulations. | ❒ | ❒ | ❒ | ❒ | ❒ |

| **Applicability of the recommendation on routine symptom monitoring** |
| --- |

| These questions are about the preliminary recommendation on symptom monitoring that you have applied in recent months.  Please tick one box per line to indicate your answer: | | | | | | | | | | | | | |
| --- | --- | --- | --- | --- | --- | --- | --- | --- | --- | --- | --- | --- | --- |
|  |  | | Very successful | | Successful | | Neutral | | Did not succeed | | Did not succeed at all | |  |
|  | | | | | | | | | | | | | |
| 38. | | To what extent did you succeed in identifying patients with incurable cancer with a life expectancy of <1 year using the surprise question? | | ❒ | | ❒ | | ❒ | | ❒ | | ❒ | |
| 39. | | To which extent did you succeed in focusing on symptom monitoring and symptom treatment on the four dimensions (physical, psychological, social and spiritual) in these patients? | | ❒ | | ❒ | | ❒ | | ❒ | | ❒ | |
| 40. | | To what extent did you succeed in recording functioning on these four dimensions and treatment agreements in the EMR for these patients? | | ❒ | | ❒ | | ❒ | | ❒ | | ❒ | |
| 41. | | To what extent did you succeed in recording these four dimensions and treatment arrangements in the EMR? | | ❒ | | ❒ | | ❒ | | ❒ | | ❒ | |

| **Determinants related to provisional recommendation** | | | | | | |
| --- | --- | --- | --- | --- | --- | --- |
| The questions below are questions of the Measurement Instrument for Determinants of Innovations (MIDI) (see: <https://www.tno.nl/media/6077/fleuren_et_al_midi_measurement_instrument.pdf>). They are about the procedural clarity, correctness, completeness, complexity, compatibility, observability and relevance for patients of the provisional recommendation on symptom monitoring that you have applied in recent months.  Please tick one box per line to indicate your answer: | | | | | | |
|  |  | Completely disagree | Disagree | Neither disagree, nor agree | Agree | Completely agree |
| 42. | The recommendation clearly describes the activities I should perform and in which order. | ❒ | ❒ | ❒ | ❒ | ❒ |
| 43. | The recommendation is based on factually correct knowledge. | ❒ | ❒ | ❒ | ❒ | ❒ |
| 44. | The recommendation provides all the information and materials needed to work with it properly. | ❒ | ❒ | ❒ | ❒ | ❒ |
| 45. | The recommendation is too complex for me to use. | ❒ | ❒ | ❒ | ❒ | ❒ |
| 46. | The recommendation is a good match for how I am used to working. | ❒ | ❒ | ❒ | ❒ | ❒ |
| 47. | The outcomes of applying the recommendation are clearly observable. | ❒ | ❒ | ❒ | ❒ | ❒ |
| 48. | I think the recommendation is relevant for my clients. | ❒ | ❒ | ❒ | ❒ | ❒ |

| **Determinants associated with the user** | | | | | | | | | | | | | | | | | | | | | |
| --- | --- | --- | --- | --- | --- | --- | --- | --- | --- | --- | --- | --- | --- | --- | --- | --- | --- | --- | --- | --- | --- |
| The questions below are questions of the Measurement Instrument for Determinants of Innovations (MIDI) (see: <https://www.tno.nl/media/6077/fleuren_et_al_midi_measurement_instrument.pdf>). They are about the benefit to you as a healthcare provider and the outcomes for your patients when applying the provisional recommendation on symptom monitoring. | | | | | | | | | | | | | | | | | | | | | |
| Please tick one box per line to indicate your answer: | | | | | | | | | | | | | | | | | | | | | |
|  | |  | | | | | Completely disagree | | | Disagree | | | Neither disagree, nor agree | | | Agree | | | Completely agree | | |
|  | |  | | | | |  | | |  | | |  | | |  | | |  | | |
| 49. | | Applying this recommendation in daily clinical practice benefits me. | | | | | ❒ | | | ❒ | | | ❒ | | | ❒ | | | ❒ | | |
| 50a. | | I think it is important to achieve the following objectives for my patients: the timely integration of palliative care in oncology. | | | | | ❒ | | | ❒ | | | ❒ | | | ❒ | | | ❒ | | |
|  | |  | | | | |  | | |  | | |  | | |  | | |  | | |
|  | |  | | | | | Most definitely not | | | Definitely not | | | Maybe | | | Definitely | | | Most definitely | | |
| 50b. | | I expect that applying the recommendation will actually achieve the following objectives for my patients: the timely integration of palliative care in oncology. | | | | | ❒ | | | ❒ | | | ❒ | | | ❒ | | | ❒ | | |
|  | |  | | | | |  | | |  | | |  | | |  | | |  | | |
|  | |  | | | | | Completely disagree | | | Disagree | | | Neither disagree, nor agree | | | Agree | | | Completely agree | | |
| 51. | | I feel it is my responsibility as a professional to apply this recommendation. | | | | | ❒ | | | ❒ | | | ❒ | | | ❒ | | | ❒ | | |
| 52. | | Patients will generally be satisfied if I apply this recommendation. | | | | | ❒ | | | ❒ | | | ❒ | | | ❒ | | | ❒ | | |
| 53. | | Patients will generally cooperate if I apply this recommendation. | | | | | ❒ | | | ❒ | | | ❒ | | | ❒ | | | ❒ | | |
| 54. | | I can count on adequate assistance from my colleagues if I need it to | | | | | ❒ | | | ❒ | | | ❒ | | | ❒ | | | ❒ | | |
|  | |  | | | | |  | | |  | | |  | | |  | | |  | | |
|  |  | | | No colleagues | Almost no colleagues | | | A minority | | | Half of my colleagues | | | A majority | | | Almost all colleagues | | | All colleagues | |
| 55. | In your opinion, what proportion of the colleagues in your organisation for whom the recommendation is intended will actually apply the recommendation? | | | ❒ | ❒ | | | ❒ | | | ❒ | | | ❒ | | | ❒ | | | ❒ | |
|  |  | | |  |  | | |  | | |  | | |  | | |  | | |  | |
|  | |  | | | | | Most definitely not | | | Definitely not | | | Maybe | | | Definitely | | | Most definitely | | |
| 56. | | Should you wish to do so, do you think you can apply the recommendation in your daily clinical practice? | | | | | ❒ | | | ❒ | | | ❒ | | | ❒ | | | ❒ | | |
|  | |  | | | | | Completely disagree | | | Disagree | | | Neither disagree, nor agree | | | Agree | | | Completely agree | | |
| 57. | | I know enough to apply this recommendation. | | | | | ❒ | | | ❒ | | | ❒ | | | ❒ | | | ❒ | | |
|  | |  | | | | |  | | |  | | |  | | |  | | |  | | |
| 58. | | To what extent are you informed about the content of the recommendation? | | | | | ❒ | | | ❒ | | | ❒ | | | ❒ | | | ❒ | | |
|  | |  | | | | |  | | |  | | |  | | |  | | |  | | |
| **Determinants associated with the organisation** | | | | | | | | | | | | | | | | | | | | |  |
| The questions below are questions of the Measurement Instrument for Determinants of Innovations (MIDI) (see: <https://www.tno.nl/media/6077/fleuren_et_al_midi_measurement_instrument.pdf>). They are about your hospital's organisation in applying the recommendation on symptom monitoring.  Please tick one box per line to indicate your answer: | | | | | | | | | | | | | | | | | | | | |  |
|  | | |  | | |  | | |  | | |  | | |  | | |  | | |  |
|  | | |  | | | Yes | | | No | | |  | | |  | | |  | | |  |
| 59. | | | Has the management set up formal arrangements in your hospital relating to applying this recommendation (in policy plans, work plans and so on)? | | | ❒ | | | ❒ | | |  | | |  | | |  | | |  |
|  | | |  | | |  | | |  | | |  | | |  | | |  | | |  |
|  | | |  | | | Completely disagree | | | Disagree | | | Neither disagree, nor agree | | | Agree | | | Completely agree | | |  |
| 60. | | | In my hospital, there are arrangements in place so that staff who apply the recommendation and leave the hospital are replaced in good time by employees who are/will be adequately prepared to take over | | | ❒ | | | ❒ | | | ❒ | | | ❒ | | | ❒ | | |  |
| 61. | | | There are enough people in our hospital to apply the recommendation as intended. | | | ❒ | | | ❒ | | | ❒ | | | ❒ | | | ❒ | | |  |
| 62. | | | There are enough financial resources available to apply the recommendation as intended. | | | ❒ | | | ❒ | | | ❒ | | | ❒ | | | ❒ | | |  |
| 63. | | | Our hospital provides me with enough time to apply the recommendation as intended in my day-to-day work. | | | ❒ | | | ❒ | | | ❒ | | | ❒ | | | ❒ | | |  |
| 64. | | | Our hospital provides me with enough materials and other resources or facilities necessary for applying the recommendation as intended | | | ❒ | | | ❒ | | | ❒ | | | ❒ | | | ❒ | | |  |
|  | | |  | | | Yes | | | No | | |  | | |  | | |  | | |  |
| 65. | | | In my hospital, one or more people have been designated to coordinate the process of applying the recommendation. | | | ❒ | | | ❒ | | |  | | |  | | |  | | |  |
|  | | |  | | |  | | |  | | |  | | |  | | |  | | |  |
| 66. | | | Are there, in addition to the implementation of the recommendation, any other changes in the hospital affecting the implementation of the recommendation now or in the foreseeable future (reorganisation, merger, cuts, staffing changes, other recommendations)? | | | ❒ | | | ❒ | | |  | | |  | | |  | | |  |
|  | | |  | | |  | | |  | | |  | | |  | | |  | | |  |
|  | | |  | | | Completely disagree | | | Disagree | | | Neither disagree, nor agree | | | Agree | | | Completely agree | | |  |
| 67. | | | It is easy for me to find information in my hospital about applying the recommendation as intended. | | | ❒ | | | ❒ | | | ❒ | | | ❒ | | | ❒ | | |  |
| 68. | | | In my hospital, feedback is regularly provided about progress with the implementation of the recommendation | | | ❒ | | | ❒ | | | ❒ | | | ❒ | | | ❒ | | |  |

| **Determinants associated with the socio-political context** | | | | | | |
| --- | --- | --- | --- | --- | --- | --- |
| The question below is a question of the Measurement Instrument for Determinants of Innovations (MIDI) (see: <https://www.tno.nl/media/6077/fleuren_et_al_midi_measurement_instrument.pdf>). It is about laws and regulations for the recommendation on symptom monitoring.  Please tick one box per line to indicate your answer: | | | | | | |
| 69. | The activities listed in the recommendation fit in well with existing legislation and regulations. | ❒ | ❒ | ❒ | ❒ | ❒ |

| **Applicability of the recommendation on the specialist palliative care team** |
| --- |

| These questions are about the preliminary recommendation on the specialist palliative care team that you have applied in recent months.  Please tick one box per line to indicate your answer: | | | | | | |
| --- | --- | --- | --- | --- | --- | --- |
|  |  | Very successful | Successful | Neutral | Did not succeed | Did not succeed at all |
|  | | | | | | |
| 70. | To what extent have you been successful in considering involving the specialist palliative care team in complex issues, such as symptom burden on multiple dimensions? | ❒ | ❒ | ❒ | ❒ | ❒ |
| 71. | To what extent have you been successful in considering involving the specialist palliative care team if the patient wishes? | ❒ | ❒ | ❒ | ❒ | ❒ |
| 72. | To what extent did you succeed in identifying patients with incurable cancer and a life expectancy of <3 months using the surprise question? | ❒ | ❒ | ❒ | ❒ | ❒ |
| 73. | To what extent did you succeed in offering these patients a discussion with the specialist palliative care team? | ❒ | ❒ | ❒ | ❒ | ❒ |

| **Determinants related to provisional recommendation** | | | | | | |
| --- | --- | --- | --- | --- | --- | --- |
| The questions below are questions of the Measurement Instrument for Determinants of Innovations (MIDI) (see: <https://www.tno.nl/media/6077/fleuren_et_al_midi_measurement_instrument.pdf>). They are about the procedural clarity, correctness, completeness, complexity, compatibility, observability and relevance for patients of the provisional recommendation on proactive care planning that you have applied in recent months.  Please tick one box per line to indicate your answer: | | | | | | |
|  |  | Completely disagree | Disagree | Neither disagree, nor agree | Agree | Completely agree |
| 74. | The recommendation clearly describes the activities I should perform and in which order. | ❒ | ❒ | ❒ | ❒ | ❒ |
| 75. | The recommendation is based on factually correct knowledge. | ❒ | ❒ | ❒ | ❒ | ❒ |
| 76. | The recommendation provides all the information and materials needed to work with it properly. | ❒ | ❒ | ❒ | ❒ | ❒ |
| 77. | The recommendation is too complex for me to use. | ❒ | ❒ | ❒ | ❒ | ❒ |
| 78. | The recommendation is a good match for how I am used to working. | ❒ | ❒ | ❒ | ❒ | ❒ |
| 79. | The outcomes of applying the recommendation are clearly observable. | ❒ | ❒ | ❒ | ❒ | ❒ |
| 80. | I think the recommendation is relevant for my clients. | ❒ | ❒ | ❒ | ❒ | ❒ |

| **Determinants associated with the user** | | | | | | | | | | | | | | |
| --- | --- | --- | --- | --- | --- | --- | --- | --- | --- | --- | --- | --- | --- | --- |
| The questions below are questions of the Measurement Instrument for Determinants of Innovations (MIDI) (see: <https://www.tno.nl/media/6077/fleuren_et_al_midi_measurement_instrument.pdf>). They are about the benefit to you as a healthcare provider and the outcomes for your patients when applying the provisional recommendation on proactive care planning. | | | | | | | | | | | | | | |
| Please tick one box per line to indicate your answer: | | | | | | | | | | | | | | |
|  | |  | | | Completely disagree | | Disagree | | Neither disagree, nor agree | | Agree | | Completely agree | |
|  | |  | | |  | |  | |  | |  | |  | |
| 81. | | Applying this recommendation in daily clinical practice benefits me. | | | ❒ | | ❒ | | ❒ | | ❒ | | ❒ | |
| 82a. | | I think it is important to achieve the following objectives for my patients: the timely integration of palliative care in oncology. | | | ❒ | | ❒ | | ❒ | | ❒ | | ❒ | |
|  | |  | | |  | |  | |  | |  | |  | |
|  | |  | | | Most definitely not | | Definitely not | | Maybe | | Definitely | | Most definitely | |
| 82b. | | I expect that applying the recommendation will actually achieve the following objectives for my patients: the timely integration of palliative care in oncology. | | | ❒ | | ❒ | | ❒ | | ❒ | | ❒ | |
|  | |  | | | Completely disagree | | Disagree | | Neither disagree, nor agree | | Agree | | Completely agree | |
| 83. | | I feel it is my responsibility as a professional to apply this recommendation. | | | ❒ | | ❒ | | ❒ | | ❒ | | ❒ | |
| 84. | | Patients will generally be satisfied if I apply this recommendation. | | | ❒ | | ❒ | | ❒ | | ❒ | | ❒ | |
| 85. | | Patients will generally cooperate if I apply this recommendation. | | | ❒ | | ❒ | | ❒ | | ❒ | | ❒ | |
| 86. | | I can count on adequate assistance from my colleagues if I need it to | | | ❒ | | ❒ | | ❒ | | ❒ | | ❒ | |
|  | |  | | |  | |  | |  | |  | |  | |
|  |  | | No colleagues | Almost no colleagues | | A minority | | Half of my colleagues | | A majority | | Almost all colleagues | | All colleagues |
| 87. | In your opinion, what proportion of the colleagues in your organisation for whom the recommendation is intended will actually apply the recommendation? | | ❒ | ❒ | | ❒ | | ❒ | | ❒ | | ❒ | | ❒ |
|  |  | |  |  | |  | |  | |  | |  | |  |
|  | |  | | | Most definitely not | | Definitely not | | Maybe | | Definitely | | Most definitely | |
| 88. | | Should you wish to do so, do you think you can apply the recommendation in your daily clinical practice? | | | ❒ | | ❒ | | ❒ | | ❒ | | ❒ | |
|  | |  | | | Completely disagree | | Disagree | | Neither disagree, nor agree | | Agree | | Completely agree | |
| 89. | | I know enough to apply this recommendation. | | | ❒ | | ❒ | | ❒ | | ❒ | | ❒ | |
|  | |  | | |  | |  | |  | |  | |  | |
| 90. | | To what extent are you informed about the content of the recommendation? | | | ❒ | | ❒ | | ❒ | | ❒ | | ❒ | |
|  | |  | | |  | |  | |  | |  | |  | |

| **Determinants associated with the organisation** | | | | | | |
| --- | --- | --- | --- | --- | --- | --- |
| The questions below are questions of the Measurement Instrument for Determinants of Innovations (MIDI) (see: <https://www.tno.nl/media/6077/fleuren_et_al_midi_measurement_instrument.pdf>). They are about your hospital's organisation in applying the recommendation on the specialist palliative care team.  Please tick one box per line to indicate your answer: | | | | | | |
|  |  |  |  |  |  |  |
|  |  | Yes | No |  |  |  |
| 91. | Has the management set up formal arrangements in your hospital relating to applying this recommendation (in policy plans, work plans and so on)? | ❒ | ❒ |  |  |  |
|  |  |  |  |  |  |  |
|  |  | Completely disagree | Disagree | Neither disagree, nor agree | Agree | Completely agree |
| 92. | In my hospital, there are arrangements in place so that staff who apply the recommendation and leave the hospital are replaced in good time by employees who are/will be adequately prepared to take over | ❒ | ❒ | ❒ | ❒ | ❒ |
| 93. | There are enough people in our hospital to apply the recommendation as intended. | ❒ | ❒ | ❒ | ❒ | ❒ |
| 94. | There are enough financial resources available to apply the recommendation as intended. | ❒ | ❒ | ❒ | ❒ | ❒ |
| 95. | Our hospital provides me with enough time to apply the recommendation as intended in my day-to-day work. | ❒ | ❒ | ❒ | ❒ | ❒ |
|  |  |  |  |  |  |  |
|  |  |  |  |  |  |  |
|  |  | Completely disagree | Disagree | Neither disagree, nor agree | Agree | Completely agree |
|  |  |  |  |  |  |  |
| 96. | Our hospital provides me with enough materials and other resources or facilities necessary for applying the recommendation as intended | ❒ | ❒ | ❒ | ❒ | ❒ |
|  |  |  |  |  |  |  |
|  |  | Ja | Nee |  |  |  |
| 97. | In my hospital, one or more people have been designated to coordinate the process of applying the recommendation. | ❒ | ❒ |  |  |  |
| 98. | Are there, in addition to the implementation of the recommendation, any other changes in the hospital affecting the implementation of the recommendation now or in the foreseeable future (reorganisation, merger, cuts, staffing changes, other recommendations)? | ❒ | ❒ |  |  |  |
|  |  |  |  |  |  |  |
|  |  | Completely disagree | Disagree | Neither disagree, nor agree | Agree | Completely agree |
| 99. | It is easy for me to find information in my hospital about applying the recommendation as intended. | ❒ | ❒ | ❒ | ❒ | ❒ |
| 100. | In my hospital, feedback is regularly provided about progress with the implementation of the recommendation | ❒ | ❒ | ❒ | ❒ | ❒ |

| **Determinants associated with the socio-political context** | | | | | | |
| --- | --- | --- | --- | --- | --- | --- |
| The question below is a question of the Measurement Instrument for Determinants of Innovations (MIDI) (see: <https://www.tno.nl/media/6077/fleuren_et_al_midi_measurement_instrument.pdf>). It is about laws and regulations for the recommendation on the specialist palliative care team.  Please tick one box per line to indicate your answer: | | | | | | |
| 101. | The activities listed in the recommendation fit in well with existing legislation and regulations. | ❒ | ❒ | ❒ | ❒ | ❒ |

**Supplementary material 2 Questionnaire for patients assessing patients experience with received care**

**Questionnaire on quality of life and quality of care**

**For patients with advanced cancer**

Please complete the questionnaire yourself. You can answer by ticking the box that best suits you. When in doubt, give the answer that best reflects your situation. There are no right or wrong answers. Although some questions may look similar, every question is different. You may find that some questions seem redundant or do not actually apply to you. We would like to urge you to still try to answer all the questions. The questionnaire is extensive because we want to capture your experiences as well as possible. If completing the questionnaire is too time-consuming or tiring for you, please try to divide it over two days.

Please return the questionnaire within **2 weeks** using the return envelope.

The answers to the questionnaire will be treated confidentially and used anonymously.

| Datum   \|  \|  \| - \|  \|  \| - \|  \|  \|  \|  \| \| --- \| --- \| --- \| --- \| --- \| --- \| --- \| --- \| --- \| --- \|   Questionnairenumber  Setting:     \|  \|  \|  \|  \|  \|  \|  \|  \|  \|  \| Wave: \| \| --- \| --- \| --- \| --- \| --- \| --- \| --- \| --- \| --- \| --- \| --- \| |
| --- | --- | --- | --- | --- | --- | --- | --- | --- | --- | --- | --- | --- | --- | --- | --- | --- | --- | --- | --- | --- | --- |

**Instructions**

- Complete the questionnaire with a black or blue pen (not a felt-tip pen).
- Put a clear cross in the answer box.
- If you fill in a wrong answer, leave the wrong cross and colour the right box completely black.
- Tick one box for each question.

| **General questions** |
| --- |

| 1. | What is your date of birth: |  |  | / |  |  | / |  |  |  |  |
| --- | --- | --- | --- | --- | --- | --- | --- | --- | --- | --- | --- |

2. What is your gender?

| ❒ | Male |  |
| --- | --- | --- |
| ❒ | Female |  |
| ❒ | Other |  |

3. What is your current marital status?

| ❒ | In a relationship, married and living together |
| --- | --- |
| ❒ | In a relationship, not married and living together |
| ❒ | In a relationship, not living together |
| ❒ | Widowed / widower / partner deceased |
| ❒ | Single / no relationship |

4. What is the highest level of education you have completed?

| ❒ | No education or primary education | |
| --- | --- | --- |
| ❒ | Secondary education | |
| ❒ | Secondary (vocational) education, or equivalent | |
| ❒ | HBO bachelor, wo bachelor | |
| ❒ | HBO master, wo master, PhD | |
| ❒ | Other, namely… | |
|  |  |  |

| **Your medical situation** |
| --- |

We are interested in certain things about you and your medical situation.

5. What type of cancer do you have?

| ❒ | Lung cancer |
| --- | --- |
| ❒ | Breast cancer |
| ❒ | Colorectal |
| ❒ | Prostate cancer |
| ❒ | Other, namely… |
|  |  |
|  |  |

| **Quality of care** |
| --- |

We are interested in some things about you and your health. Please answer all of the questions yourself by circling the number that best applies to you. There are no "right" or "wrong" answers. The information that you provide will remain strictly confidential. These questions below are of the EORTC-INPATSAT (see: <https://www.eortc.org/app/uploads/sites/2/2018/08/Specimen-IN-PATSAT32-English.pdf>)

| **During your hospital stay, how would Poor Fair Good Very Excellent you rate doctors, in terms of:** | | *Poor* | *Fair* | *Good* | *Very good* | *excellent* |
| --- | --- | --- | --- | --- | --- | --- |
|  | | **1** | **2** | **3** | **4** | **5** |
| 6. | Their knowledge and experience of your illness? | ❒ | ❒ | ❒ | ❒ | ❒ |
| 7. | The treatment and medical follow-up they provided? | ❒ | ❒ | ❒ | ❒ | ❒ |
| 8. | The attention they paid to your physical problems? | ❒ | ❒ | ❒ | ❒ | ❒ |
| 9. | Their willingness to listen to all of your concerns? | ❒ | ❒ | ❒ | ❒ | ❒ |
| 10. | The interest they showed in you personally? | ❒ | ❒ | ❒ | ❒ | ❒ |
| 11. | The comfort and support they gave you? | ❒ | ❒ | ❒ | ❒ | ❒ |
| 12. | The empathy they showed? | ❒ | ❒ | ❒ | ❒ | ❒ |
| 13. | The information they gave you about your illness? | ❒ | ❒ | ❒ | ❒ | ❒ |
| 14. | The information they gave you about your medical tests or treatment? | ❒ | ❒ | ❒ | ❒ | ❒ |
| 15. | The time they devoted to you during visits/consultations? | ❒ | ❒ | ❒ | ❒ | ❒ |
| 16. | How would you rate the care received this past month | ❒ | ❒ | ❒ | ❒ | ❒ |

During the past month:

17. Was there a good match between the care provided by the different caregivers involved in looking after you?

| ❒ | Never |
| --- | --- |
| ❒ | Sometimes |
| ❒ | Most of the time |
| ❒ | Always |

18. Do healthcare providers give you contradictory information?

| ❒ | Never |
| --- | --- |
| ❒ | Sometimes |
| ❒ | Most of the time |
| ❒ | Always |

| **Your health** |
| --- |

We are interested in some things about you and your health. The questions are about your own health during the past week. These questions below are of the EORTC-QLQ-C30

(see: <https://www.eortc.org/app/uploads/sites/2/2018/08/Specimen-QLQ-C30-English.pdf>)

|  | | | *Not at all* | | *A little* | | *Quite a bit* | | *Very much* | |  |
| --- | --- | --- | --- | --- | --- | --- | --- | --- | --- | --- | --- |
|  | | | **1** | | **2** | | **3** | | **4** | |  |
| 19. | | Do you have any trouble doing strenuous activities, like carrying a heavy shopping bag or a suitcase? | | ❒ | | ❒ | | ❒ | | ❒ | |
| 20. | | Do you have any trouble taking a long walk? | | ❒ | | ❒ | | ❒ | | ❒ | |
| 21. | | Do you have any trouble taking a short walk outside of the house? | | ❒ | | ❒ | | ❒ | | ❒ | |
| 22. | | Do you need to stay in bed or a chair during the day? | | ❒ | | ❒ | | ❒ | | ❒ | |
| 23. | | Do you need help with eating, dressing, washing yourself or using the toilet? | | ❒ | | ❒ | | ❒ | | ❒ | |
|  |  | |  | |  | |  | |  | |  |

During the past week:

|  | | *Not at all* | *A little* | *Quite a bit* | *Very much* |
| --- | --- | --- | --- | --- | --- |
|  | | **1** | **2** | **3** | **4** |
| 24. | Were you short of breath? | ❒ | ❒ | ❒ | ❒ |
| 25. | Have you had pain? | ❒ | ❒ | ❒ | ❒ |
| 26. | Did you need to rest? | ❒ | ❒ | ❒ | ❒ |
| 27. | Have you had trouble sleeping? | ❒ | ❒ | ❒ | ❒ |
| 28. | Have you felt weak? | ❒ | ❒ | ❒ | ❒ |
| 29. | Have you lacked appetite? | ❒ | ❒ | ❒ | ❒ |
| 30. | Have you felt nauseated? | ❒ | ❒ | ❒ | ❒ |
| 31. | Have you vomited? | ❒ | ❒ | ❒ | ❒ |
| 32. | Have you been constipated? | ❒ | ❒ | ❒ | ❒ |
| 33. | Have you had diarrhea? | ❒ | ❒ | ❒ | ❒ |
| 34. | Were you tired? | ❒ | ❒ | ❒ | ❒ |
| 35. | Did you feel tense? | ❒ | ❒ | ❒ | ❒ |
| 36. | Did you worry? | ❒ | ❒ | ❒ | ❒ |
| 37. | Did you feel irritable? | ❒ | ❒ | ❒ | ❒ |
| 38. | Did you feel depressed? | ❒ | ❒ | ❒ | ❒ |
|  |  |  |  |  |  |
| 39. | Were you limited in doing either your work or other daily activities? | ❒ | ❒ | ❒ | ❒ |
| 40. | Were you limited in pursuing your hobbies or other leisure time activities? | ❒ | ❒ | ❒ | ❒ |
| 41. | Did pain interfere with your daily activities? | ❒ | ❒ | ❒ | ❒ |
| 42. | Have you had difficulty in concentrating on things, like reading a newspaper or watching television? | ❒ | ❒ | ❒ | ❒ |
| 43. | Have you had difficulty remembering things? | ❒ | ❒ | ❒ | ❒ |
| 44. | Has your physical condition or medical treatment interfered with your family life? | ❒ | ❒ | ❒ | ❒ |
| 45. | Has your physical condition or medical treatment interfered with your social activities? | ❒ | ❒ | ❒ | ❒ |
| 46. | Has your physical condition or medical treatment caused you financial difficulties? | ❒ | ❒ | ❒ | ❒ |

For the following questions please circle the number between 1 and 7 that best applies to you

47. How would you rate your overall health during the past week?

| *Very poor* | |  | | | | | *Excellent* | |
| --- | --- | --- | --- | --- | --- | --- | --- | --- |
| 1 | 2 | | 3 | 4 | 5 | 6 | | 7 |
| ❒ | ❒ | | ❒ | ❒ | ❒ | ❒ | | ❒ |

48. How would you rate your overall quality of life during the past week?

| *Very poor* | |  | | | | | *Excellent* | |
| --- | --- | --- | --- | --- | --- | --- | --- | --- |
| 1 | 2 | | 3 | 4 | 5 | 6 | | 7 |
| ❒ | ❒ | | ❒ | ❒ | ❒ | ❒ | | ❒ |

These questions below are of the FACIT-SP (see: <https://www.facit.org/measure-english-downloads/facit-sp-english-downloads>). Please circle or mark one number per line to indicate your response as it applies to the past 7 days.

|  | | *Not at all* | | *A little bit* | *Somewhat* | *Quite a bit* | | *Very much* | | |
| --- | --- | --- | --- | --- | --- | --- | --- | --- | --- | --- |
| 49. | I feel peaceful | | ❒ | ❒ | ❒ | | ❒ | | ❒ |  |
| 50. | I have a reason for living | | ❒ | ❒ | ❒ | | ❒ | | ❒ |  |
| 51. | My life has been productive | | ❒ | ❒ | ❒ | | ❒ | | ❒ |  |
| 52. | I have trouble feeling peace of mind | | ❒ | ❒ | ❒ | | ❒ | | ❒ |  |
| 53. | I feel a sense of purpose in my life | | ❒ | ❒ | ❒ | | ❒ | | ❒ |  |
| 54. | I am able to reach down deep into myself for comfort | | ❒ | ❒ | ❒ | | ❒ | | ❒ |  |
| 55. | I feel a sense of harmony within myself | | ❒ | ❒ | ❒ | | ❒ | | ❒ |  |
| 56. | My life lacks meaning and purpose | | ❒ | ❒ | ❒ | | ❒ | | ❒ |  |
| 57. | I find comfort in my faith or spiritual beliefs | | ❒ | ❒ | ❒ | | ❒ | | ❒ |  |
| 58. | I find strength in my faith or spiritual beliefs | | ❒ | ❒ | ❒ | | ❒ | | ❒ |  |
| 59. | My illness has strengthened my faith or spiritual beliefs | | ❒ | ❒ | ❒ | | ❒ | | ❒ |  |
| 60. | I know that whatever happens with my illness, things will be okay | | ❒ | ❒ | ❒ | | ❒ | | ❒ |  |

| **Received care** |
| --- |
| We are interested in some things about you and your health. The questions are about your own health during since your diagnosis. |

| 61. | Did you discuss your goals, choices and what care suits them now and in the future with a clinician? |
| --- | --- |
| ❒ | Yes |
| ❒ | No → Proceed to question 65 |

Please tick one box to indicate your response

|  | | *Not at all* | | *A little* | *Somewhat* | *To a fairly high degree* | | *To a very high degree* | | |
| --- | --- | --- | --- | --- | --- | --- | --- | --- | --- | --- |
| 62. | To what extent did you find it pleasant? | | ❒ | ❒ | ❒ | | ❒ | | ❒ |  |
| 63. | To what extent did you find it helpful? | | ❒ | ❒ | ❒ | | ❒ | | ❒ |  |
| 64. | To what extent are you content with it? | | ❒ | ❒ | ❒ | | ❒ | | ❒ |  |

Room for comments:

|  |  |
| --- | --- |
|  |  |

| 65. | Did a clinician ask about your physical problems? |
| --- | --- |
| ❒ | Yes |
| ❒ | No → Proceed to question 69 |

Please tick one box to indicate your response

|  | | *Not at all* | | *A little* | *Somewhat* | *To a fairly high degree* | | *To a very high degree* | | |
| --- | --- | --- | --- | --- | --- | --- | --- | --- | --- | --- |
| 66. | To what extent did you find it pleasant? | | ❒ | ❒ | ❒ | | ❒ | | ❒ |  |
| 67. | To what extent did you find it helpful? | | ❒ | ❒ | ❒ | | ❒ | | ❒ |  |
| 68. | To what extent are you content with it? | | ❒ | ❒ | ❒ | | ❒ | | ❒ |  |

Room for comments:

|  |  |
| --- | --- |
|  |  |

| 69. | Did a clinician ask about your psychological/emotional problems? |
| --- | --- |
| ❒ | Yes |
| ❒ | No → Proceed to question 73 |

Please tick one box to indicate your response

|  | | *Not at all* | | *A little* | *Somewhat* | *To a fairly high degree* | | *To a very high degree* | | |
| --- | --- | --- | --- | --- | --- | --- | --- | --- | --- | --- |
| 70. | To what extent did you find it pleasant? | | ❒ | ❒ | ❒ | | ❒ | | ❒ |  |
| 71. | To what extent did you find it helpful? | | ❒ | ❒ | ❒ | | ❒ | | ❒ |  |
| 72. | To what extent are you content with it? | | ❒ | ❒ | ❒ | | ❒ | | ❒ |  |

Room for comments:

|  |  |
| --- | --- |
|  |  |

| 73. | Did a clinician ask about your social problems? |
| --- | --- |
| ❒ | Yes |
| ❒ | No → Proceed to question 77 |

Please tick one box to indicate your response

|  | | *Not at all* | | *A little* | *Somewhat* | *To a fairly high degree* | | *To a very high degree* | | |
| --- | --- | --- | --- | --- | --- | --- | --- | --- | --- | --- |
| 74. | To what extent did you find it pleasant? | | ❒ | ❒ | ❒ | | ❒ | | ❒ |  |
| 75. | To what extent did you find it helpful? | | ❒ | ❒ | ❒ | | ❒ | | ❒ |  |
| 76. | To what extent are you content with it? | | ❒ | ❒ | ❒ | | ❒ | | ❒ |  |

Room for comments:

|  |  |
| --- | --- |
|  |  |

| 77. | Did a clinician ask about your spiritual challenges? |
| --- | --- |
| ❒ | Yes |
| ❒ | No → Proceed to question 81 |
|  |  |

Please tick one box to indicate your response

|  | | *Not at all* | | *A little* | *Somewhat* | *To a fairly high degree* | | *To a very high degree* | | |
| --- | --- | --- | --- | --- | --- | --- | --- | --- | --- | --- |
| 78. | To what extent did you find it pleasant? | | ❒ | ❒ | ❒ | | ❒ | | ❒ |  |
| 79. | To what extent did you find it helpful? | | ❒ | ❒ | ❒ | | ❒ | | ❒ |  |
| 80. | To what extent are you content with it? | | ❒ | ❒ | ❒ | | ❒ | | ❒ |  |

Room for comments:

|  |  |
| --- | --- |
|  |  |

| 81. | Were you informed about the possibility of having a consultation with a PC consultant? |
| --- | --- |
| ❒ | Yes |
| ❒ | No → skip questions 82 until 88 |

Please tick one box to indicate your response

|  | | *Not at all* | | *A little* | *Somewhat* | *To a fairly high degree* | | *To a very high degree* | | |
| --- | --- | --- | --- | --- | --- | --- | --- | --- | --- | --- |
| 82. | To what extent did you find it pleasant? | | ❒ | ❒ | ❒ | | ❒ | | ❒ |  |
| 83. | To what extent did you find it helpful? | | ❒ | ❒ | ❒ | | ❒ | | ❒ |  |
| 84. | To what extent are you content with it? | | ❒ | ❒ | ❒ | | ❒ | | ❒ |  |

Room for comments:

|  |  |
| --- | --- |

| 85. | Did you have a consultation with a palliative care consultant? |
| --- | --- |
| ❒ | Yes |
| ❒ | No → skip questions 86, 87 and 88 |

Please tick one box to indicate your response

|  | | *Not at all* | | *A little* | *Somewhat* | *To a fairly high degree* | | *To a very high degree* | | |
| --- | --- | --- | --- | --- | --- | --- | --- | --- | --- | --- |
| 86. | To what extent did you find it pleasant? | | ❒ | ❒ | ❒ | | ❒ | | ❒ |  |
| 87. | To what extent did you find it helpful? | | ❒ | ❒ | ❒ | | ❒ | | ❒ |  |
| 88. | To what extent are you content with it? | | ❒ | ❒ | ❒ | | ❒ | | ❒ |  |

Room for comments:

|  |  |
| --- | --- |

| Do you have any further comments? You can note them below, e.g. what you thought of this questionnaire or other comments. |
| --- |

|  |
| --- |

This is the end of the questionnaire. Please check that you have not skipped any questions. Please return the questionnaire within two weeks in the enclosed reply envelope. A stamp is not necessary!

Thank you very much for your cooperation in this survey.

For more information, or if you feel the need to talk further about the questions or feelings you have about completing the questionnaire, you can contact the researcher, Carly Heipon, at any time. (c.heipon@iknl.nl, 06 43 20 27 01).

| **Supplementary table 1 Implementation of recommendations per hospital** | | | | |
| --- | --- | --- | --- | --- |
|  | **Hospital 1** | **Hospital 2** | **Hospital 3** | **Hospital 4** |
| **Setting** | In- and outpatient of oncology department | In- and outpatients of oncology department | Outpatients of oncology department | In- and outpatients department of lung oncology |
| **ACP discussions for all patients with incurable cancer** |  |  |  |  |
| - How were patients informed about ACP discussion(s)? | Verbally | Verbally | Verbally and through leaflet | Verbally |
| - Who conducted the ACP discussion(s)? | Oncologist or nurse practitioner | A consultant of the SPCT | Case manager and/or consultant of the SPCT | A consultant of the SPCT |
| - How were the ACP discussion(s) recorded? | EMR | Existing format in EMR | EMR | Existing format in EMR |
| - How were the outcomes of exchanged with the primary care physician or involved institutional physician? | By phone or letter | By letter | By letter | By letter |
| **Symptom monitoring for all patients with a life expectancy of < 1 year** |  |  |  |  |
| - Who identified patients with a life expectancy of < 1 year | Oncologist | Oncology nurses | Oncologist | Case manager in coordination with treating physician |
| - Which tool was used to monitor symptoms? | USD-4 | USD-4 | USD-4 | Existing format in EMR which includes the Hospital Anxiety and Depression Scale and Distress Thermometer |
| - How often were symptoms monitored? | During every visit | During every visit, at least every 3 months | 4 times a year | At least once a month, with a maximum of once a week |
| **Consider to involve the SPCT in cases of complexity, such as symptom burden on multiple dimensions, and/or if the patient requests their involvement** |  |  |  |  |
| - How were patients identified? | Symptom burden on more than 1 dimension on the USD-4 | Symptom burden on more than 1 dimension on the USD-4 | Using the USD-4 and clinical assessment of patients functioning, problems on the psychological or existential domain | Using an existing app and clinical assessment |
| - Who identified patients? | Oncologist | Nurses |  | Treating physician and case manager |
| **Offering patients a consultation with a consultant of the SPCT for all patients with a life expectancy of <3 months** |  |  |  |  |
| - How were patients identified? | Clinical assessment |  | Clinical assessment and problems on psychological and existential domain | Clinical assessment |
| - How were patients informed about the SPCT? | Verbally |  | Verbally and through a leaflet | Verbally and through a leaflet |
| - Who offered patients a consultation with a consultant of the SPCT? | Oncologist or nurse practitioner | Nurses | Case manager in coordination with treating physician | Case manager in coordination with treating physician |

| **Supplementary table 2 Sociodemographic characteristics of clinicians (N=27)** | |
| --- | --- |
|  | **n (%)** |
| Age (mean (SD); range min-max) | 46 (10); 33-66 |
| Sex |  |
| Female | 23 (85) |
| Male | 4 (15) |
| Profession |  |
| Physician | 13 (48) |
| Nurse practitioner | 1 (4) |
| Nurse | 13 (48) |
| Additionally trained in palliative care |  |
| No | 10 (37) |
| 2-years palliative care CME^1^ for physicians | 2 (7) |
| 9-day course in palliative care for physicians | 3 (11) |
| Nurses with 1-year palliative care CNE^2^ | 3 (11) |
| Courses on palliative care | 8 (30) |
| Internship palliative care within the hospital | 1 (4) |
| ^1^ CME: Continuing medical education  ^2^ CNE: Continuing nursing education |  |

| **Supplementary table 3 Applying the recommendations in daily practice per hospital** | | | | | |
| --- | --- | --- | --- | --- | --- |
|  | **Hospital 1 (N=150)** | **Hospital 2 (N=125)** | **Hospital 3 (N=150)** | **Hospital 4 (N=117)** | **Total**  **(N=542)** |
|  | **Yes n (%)** | **Yes n (%)** | **Yes n (%)** | **Yes n (%)** | **Yes n (%)** |
| **Advance Care Planning** |  |  |  |  |  |
| Was an ACP discussion conducted in the previous year? | 96 (64) | 56 (45) | 72 (48) | 43 (37) | 267 (49) |
| If ACP was conducted: Were the outcomes of the ACP discussion shared with the GP and/or involved institutional physician? | 96 (100) | 34 (61) | 70 (97) | 24 (56) | 224 (84) |
| **Routine symptom monitoring** |  |  |  |  |  |
| A negative answer to the surprise question 12 months (‘Would I be surprised if this patient died in the next 12 months?’) | n=79 | n=61 | n=36 | n=44 | n=220 |
| If SQ 12 months is negative: Were the patients’ functioning on the four dimensions and/or the treatment agreements recorded in the EMR? | 58 (73) | 30 (49) | 20 (56) | 19 (43) | 127 (58) |
| **Involvement of SPCT** |  |  |  |  |  |
| A negative answer to the surprise question 3 months (‘Would I be surprised if this patient died in the next 3 months?’) | n=18 | n=14 | n=10 | n=23 | n=65 |
| If SQ 3 negative: Were these patients offered a consultation with the SPCT? | 13 (72) | 7 (50) | 8 (80) | 10 (83) | 38 (58) |
